# The effect of a brown-rice diets on glycemic control and metabolic parameters in prediabetes and type 2 diabetes mellitus: a meta-analysis of randomized controlled trials and controlled clinical trials

**DOI:** 10.7717/peerj.11291

**Published:** 2021-05-26

**Authors:** Anis Farhanah Abdul Rahim, Mohd Noor Norhayati, Aida Maziha Zainudin

**Affiliations:** 1 Department of Family Medicine, School of Medical Sciences, Universiti Sains Malaysia, Kubang Kerian, Kelantan, Malaysia; 2 Department of Pharmacology, School of Medical Sciences, Universiti Sains Malaysia, Kubang Kerian, Kelantan, Malaysia

**Keywords:** Brown rice, Prediabetes, Diabetes, Glycaemic control, Meta-analysis

## Abstract

**Background:**

Brown rice is a whole-grain food that is often assumed to have a lower glycemic index compared to white rice. A few studies have objectively confirmed the effect of a brown-rice diet on glycemic control and metabolic parameters compared to a white-rice diet. The purpose of this study is to determine the effect of brown rice on improving glycemic control and metabolic parameters in prediabetes and type 2 diabetes. The researchers conducted a systematic review and meta-analysis of randomized controlled trials (RCTs) and controlled clinical trials.

**Methods:**

PRISMA guidelines were used as the basis of this systematic review. Relevant studies were identified by searching the following databases: Cochrane Central Register of Controlled Trials (CENTRAL), MEDLINE (PubMed), as well as Epistemonikos for randomized controlled trials (RCTs) and controlled clinical trials published not later than January 2021 involving adults with prediabetes and diabetes mellitus who were consuming brown rice compared to those consuming white rice. The primary outcomes measured were glycated hemoglobin (HbA1c) and fasting blood glucose (FBG) levels. The secondary outcomes were body weight, waist circumference, systolic and diastolic blood pressure levels, LDL and HDL-cholesterol levels. The mean differences (MDs) with 95% confidence intervals (CIs) between brown and white-rice-diet groups were calculated using a random-effects model.

**Results:**

Seven trials involving 417 adults with prediabetes or type 2 diabetes were included in this study. Brown-rice diet did not improve the glycemic control because it had no effect on the HbA1c level (*p* = 0.15) and the FBG level (*p* = 0.95) compared to white-rice diet. Brown-rice diet reduced body weight (*p* < 0.00001; MD −2.2 kg; 95% CI [−3.13 to −1.26]; *I*^2^ = 0%). However, it had no effect on the waist circumference (*p* = 0.09), systolic blood pressure (*p* = 0.60) and diastolic blood pressure level (*p* = 0.40). HDL-cholesterol level is increased in brown-rice diet (*p* = 0.01; MD 0.10, 95% CI [0.02 to 0.17]; *I*^2^ = 44%) but it had no effect on the LDL-cholesterol level (*p* = 0.81).

**Conclusions:**

The available evidence indicated that consuming brown rice in substitute for white rice does not affect glycemic control (HbA1c and FBG levels) in pre-diabetes and type 2 diabetes patients. Brown rice, however, may be used as an alternative for white rice in such patients because it was found to reduce body weight and increase the HDL-cholesterol level. The benefits of a brown-rice diet on glycemic control may not be detected in short-term studies. The obtained evidence in this meta-analysis ranged from low to moderate quality. Thus, more high-quality trials with a larger sample size and a longer follow-up duration are needed to further investigate the effects of a brown-rice diet on diabetes glycemic control with stronger evidence.

PROSPERO registration number: CRD42019143266

## Introduction

Diabetes mellitus (DM) is a global health burden that is increasing at an alarming rate. An estimated 422 million adults lived with diabetes in 2014 globally compared to only 108 million in 1986 (Roglic, 2016). Four out of five adults with diabetes live in low- and middle- income countries (Guariguata et al., 2014; Roglic, 2016). Lifestyle modifications, which includes diet and exercise, are essential key component in the treatment of type 2 diabetes, with or without pharmacotherapy (Evert et al., 2013). The aim is to improve the quality of life and prevent diabetic complications, which can be achieved by maintaining good glycemic control (Chatterjee & Davies, 2015). The importance of dietary intervention through its effect on body weight and metabolic control are clear, but diet is also one of the most controversial and difficult aspects in the management of type 2 diabetes. Medical nutrition therapy was introduced to guide a systematic and evidence-based approach in the management of diabetes through proper diet, and its effectiveness has been demonstrated (Morris & Wylie-Rosett, 2010).

Rice is the staple food for more than half of the global population. White rice is the most commonly consumed type, but brown rice is often recommended as a healthier option to maintain good glycemic control in a rice-consuming people (Imam et al., 2012). Studies have shown that white rice, which has a high glycemic index, increases the risk of diabetes and may worsen the glycemic control in diabetic patients (Boers, Seijen Ten Hoorn & Mela, 2015; Hu et al., 2012). Panlasigui and Thompson reported that brown rice has a lower glycemic index, which generates a lower postprandial glucose response (Panlasigui & Thompson 2006). This was supported by a systematic review by Foster-Powell, Holt & Brand-Miller (2002), which found that the mean glycemic index of white rice is 64 and that of brown rice is only 55 (Foster-Powell, Holt & Brand-Miller, 2002).

Brown rice is a whole grain while white rice is a refined grain. Whole-grain intake has a protective effect on type 2 diabetes risk by decreasing the energy intake, preventing weight gain, and increasing insulin sensitivity (Aune et al., 2013; Murtaugh et al., 2003; Parker et al., 2013). A meta-analysis reported that larger whole-grain intake is associated with a lower risk of developing type 2 diabetes and cardiovascular disease and as well as a lower risk of weight gain (Ye et al., 2012). However, the studies by Ranawana et al. (2009) and Boers, Seijen Ten Hoorn & Mela (2015) indicated that the glycemic-index difference between brown and white rice is inconsistent as the milling and cooking process may affect the postprandial glucose (PPG) and insulin responses.

### Description of the intervention

Brown rice is whole-grain rice with the inedible outer hull removed. To convert brown rice into white rice, the complete milling and polishing process removes the hull, bran layer, and cereal germ, which reduces its nutritional value (Dinesh Babu, Subhasree & Rajagopal, 2009). Brown rice contains phytochemicals such as polyphenols, oryzanol, phytosterols, tocotrienols, tocopherols, and carotenoids as well as vitamins and minerals.

### Rationale

Replacing a white-rice diet with a brown-rice diet is widely assumed to benefit people with diabetes. However, limited studies have objectively confirmed the effect of a brown-rice diet on glycemic control and metabolic parameters compared to white-rice diet. The purpose of this study was to determine the effect of brown-rice diet on improving glycemic control and metabolic parameters in prediabetes and type 2 diabetes patients.

## Material and Methods

This article presents the conduct and results of a systematic review and meta-analysis of randomized controlled trials (RCTs) and controlled clinical trials, that examined the effect of a brown-rice diet on glycemic control and metabolic parameters in adults with prediabetes or type 2 diabetes compared with a white-rice diet. The research was conducted following the recommendations outlined in the PRISMA (Preferred Reporting Items for Systematic Reviews and Meta-Analyses) guidelines.

Relevant studies were identified by searching the following databases: Cochrane Central Register of Controlled Trials (CENTRAL), MEDLINE (PubMed), as well as Epistemonikos for randomized controlled trials (RCTs) and controlled clinical trials published not later than January 2021 involving adults with prediabetes or type 2 diabetes who were consuming brown rice orwhite rice. The search terms included “brown rice”, “prediabetes”, and “diabetes”, and Boolean operators such as AND, OR, truncation, and wildcards were used for variations in words. The researchers also searched for ongoing trials through the World Health Organization International Clinical Trials Registry Platform (ICTRP) and ClinicalTrials.gov. Only English language publications were considered for this study. The review protocol was submitted to PROSPERO and was registered in October 2019. The literature search was updated until January 2021.

The inclusion criteria were defined using the PICOS model. The study population consists of adults with prediabetes and type 2 diabetes, and the intervention was a brown-rice diet for more than two weeks duration, compared to a white-rice diet for the same duration. The primary outcomes were glycated hemoglobin (HbA1c) and fasting blood glucose (FBG) while the secondary outcomes were body weight, waist circumference, systolic and diastolic blood pressure levels, and serum lipid profile (HDL- and LDL-cholesterol levels). We included parallel and crossover RCTs and controlled clinical trials.

Ethical approval was not obtained because this study used data from published trials.

Two reviewers (AFAR, NMN) independently assessed the eligibility of the trials to be included in the systematic review and meta-analysis. The eligibility of the trials was assessed, and the reasons for trial exclusion were documented. Trials that met the eligibility criteria with full-text articles were obtained. Disagreements were resolved by discussion with another author (AMZ).

Two independent authors (AFAR, NMN) assessed the risk of bias using the Cochrane risk-of-bias tool for randomized trials based on random sequence generation, allocation concealment; blinding of the participants, personnel, and outcome assessors; completeness of the outcome data; selectivity of the outcome reporting; and other biases (Higgins et al., 2019). The researchers categorized the risk of bias as low, unclear, or high. Any disagreement was resolved by discussion.

The data were independently extracted by two reviewers (AFAR, NMN) using a data extraction form. The differences were resolved by discussion, and if necessary, another author (AMZ) was consulted. The following data were extracted: characteristics of the trials (study setting and duration), the participants’ characteristics (age, gender, and ethnicity), the method used for the trials (numbers of participants randomized and analyzed, duration of follow-up), description of the intervention, and the study outcomes. The primary outcomes that were monitored include the HbA1c and FBG levels. The secondary outcomes that were monitored consist of body weight, waist circumference, systolic and diastolic blood pressure levels, and serum lipid profile (HDL- and LDL-cholesterol levels).

### Statistical analysis

All the statistical analyses were carried out using the Review Manager software (Review Manager, 2011). For all the included trials with continuous outcomes, we calculated the mean differences (MDs) with 95% confidence intervals (CI). We presented the results as a summary of the risk ratios (RR) for those with dichotomous outcomes. We reported the results using the fixed-effect and random-effects models (Higgins et al., 2019), utilizing inverse variance. We pooled these measures in the meta-analysis and drew forest plots.

We assessed the findings for obvious heterogeneity by comparing the populations, interventions, comparators, and outcomes. We then assessed the statistical heterogeneity with the I^2^ statistic (Higgins et al., 2019). The different thresholds of the I2 statistics were interpreted as follows: 0–40%, may not be important; 30–60%, may indicate moderate heterogeneity; 50–90%, may indicate substantial heterogeneity; and 75–100%, may indicate considerable heterogeneity (Higgins et al., 2019).

### Quality assessment

The researchers used a GRADE approach to evaluate the quality of evidence in the systematic reviews and the strength of the recommendations. The quality of evidence was downgraded based on any of the following: (i) limitations in the study design; (ii) indirectness of the evidence; (iii) inconsistency of the results or unexplained heterogeneity; (iv) imprecision of the results; and (v) publication bias (Balshem et al., 2011; Guyatt et al., 2008). The researchers used the GRADEpro guideline development tool (2015, McMaster University and Evidence Prime, Inc.) to grade the quality of evidence for each outcome.

### Deviations from the registered protocol

There were several deviations from the registered protocol due to the use of the random- effects model for all the analyses instead of the fixed-effect or random-effects model based on heterogeneity. This was based on the recommendation in the updated version of Cochrane Handbook 2019. The review protocol was submitted to PROSPERO and was registered in October 2019. The literature search was updated until January 2021.

## Results

**Figure 1:**
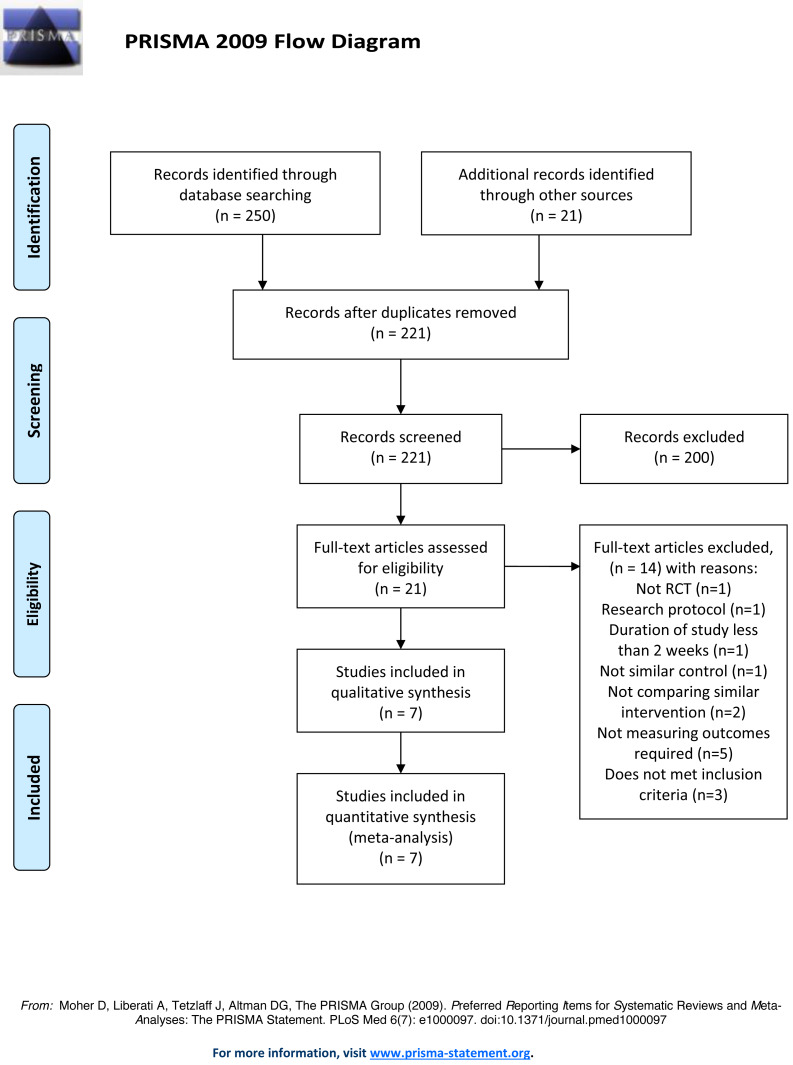
PRISMA 2009 flow diagram.

A total of 271 relevant articles were found in the electronic searches. After removing the duplicates, 221 articles were left, and these were screened. 200 articles were excluded because they did not meet the inclusion criteria based on their abstracts. Twenty-one full text articles were reviewed and assessed for eligibility (Fig. 1). The exclusions included one review article, one research protocol, two trials that did not compare similar interventions and one not comparing with similar control, one trials discussing a study with a duration of less than two weeks, five trials that did not present measurement of the outcomes required, and three trials did not meet the participant inclusion criteria. A total of seven trials met the eligibility criteria and were included in the qualitative and quantitative synthesis.

### Characteristics of the included trials

The characteristics of the seven included trials are summarized in Table 1. Four parallel RCTs (i.e., Araki et al., 2017; Kondo et al., 2017; Wang et al., 2012; Zhang et al., 2011), two crossover RCTs (i.e., Hsu et al., 2008; Nakayama et al., 2017), and one controlled clinical trial (i.e., Bui et al., 2014) that compared the effects of a brown-rice diet on individuals with prediabetes or type 2 diabetes with those of a white-rice diet were included. A total of 415 participants were involved. Three trials were on prediabetes (*n* = 158) and four trials were on type 2 diabetes (*n* = 259). All the trials involved Asian populations, particularly Vietnamese, Taiwanese, Japanese, Chinese, and Chinese Americans. There were 209 participants in the white-rice diet group, and 206 in the brown-rice diet group. The mean age of the participants was 56.2 years, and 233 of the participants were women while 182 were men.

**Table 1:** Characteristics of included trials.

**Studies**	**Size, *n***	**Study duration**	**Study population**	**Intervention**	**Comparison**
Araki, 2017	41	12 weeks	Pre-diabetic, Japanese	partially abraded brown rice, *n* = 20	white rice, *n* = 21
Bui, 2014	60	16 weeks	Pre-diabetic, Vietnamese	pre-germinated brown rice, *n* = 30	white rice, *n* = 30
Hsu, 2008	11	6 weeks	Type 2 diabetes mellitus, Taiwanese	pre-germinated brown rice, *n* = 5	white rice, *n* = 6
Kondo, 2017	28	8 weeks	Type 2 diabetes mellitus, Japanese	brown rice diet, *n* = 14	white rice, *n* = 14
Nakayama, 2017	16	8 weeks	Type 2 diabetes mellitus, Japanese	glutinous brown rice, *n* = 8	white rice, *n* = 8
Wang, 2013	57	12 weeks	Pre-diabetic, Chinese Americans	brown rice, *n* = 28	white rice, *n* = 29
Zhang, 2011	202	16 weeks	Type 2 diabetes mellitus, Chinese	brown rice, *n* = 101	white rice, *n* = 101

All the participants in the intervention group received brown rice as a staple food while maintaining normal daily life activities without restrictions. The participants in the control group received a white-rice diet. Three trials did not report the type of brown rice that were used, but two trials used pre-germinated brown rice, one trial used glutinous brown rice, and another trial used partially abraded brown rice. The intervention follow-up periods ranged from 6 to 16 weeks.

Concerning the crossover RCTs (i.e., Hsu et al., 2008; Nakayama et al., 2017), data were taken prior to crossover during the first intervention phase (Higgins et al., 2021).

### Risk-of-bias assessment

**Figure 2:**
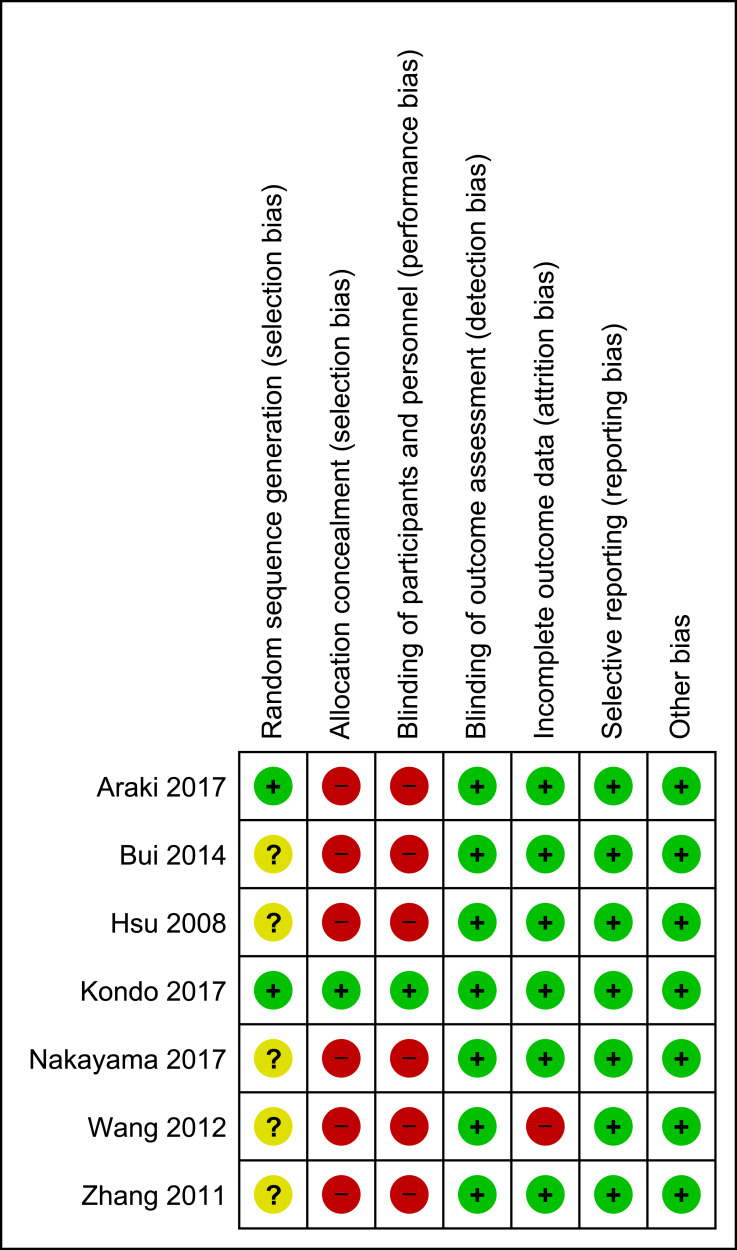
Risk of bias summary.

The assessment of the risk of bias is shown in Fig. 2. Each study was assessed as having a low, a high, or an unclear risk of bias for each of the risk-of-bias indicators. For random sequence generation, one trial was a controlled clinical trial (i.e., Bui et al., 2014), in which no randomization procedure was described; thus, it had a high risk of selection bias. Two trials (i.e., Araki et al., 2017; Kondo et al., 2017) were assessed as having a low risk of bias, as they generated a random sequence by age stratification using the minimization method. The other four trials (i.e, Hsu et al., 2008; Nakayama et al., 2017; Wang et al., 2012; Zhang et al., 2011) had an unclear risk of bias because the method of random sequence generation that was used was not mentioned. The allocation concealment and blinding of the participants had a high risk of bias given the obvious differences in appearance and texture between brown rice and white rice. Only one study (i.e., Kondo et al., 2017) was assessed as having a low risk of bias for allocation concealment because the participants were provided with both brown and white rice in small single-serve pouches while another two trials (i.e., Hsu et al., 2008; Nakayama et al., 2017) were assessed as having an unclear risk of bias because the allocation concealment method that was used was not mentioned. Six of the seven included trials did not state the blinding of the outcome assessors (i.e., Araki et al., 2017; Bui et al., 2014; Kondo et al., 2017; Wang et al., 2012; Zhang et al., 2011), but it was unlikely that the trial outcomes were influenced by the fact that the assessors were not blinded because the outcomes were objective assessments of laboratory results and anthropometric measurements. Therefore, the blinding of the outcome assessors was posed as a low risk of bias. The risk of attrition bias was low in six trials. One trial was assessed as having a high risk of attrition bias because it reported discrepant numbers in the text and tables and failed to report the intention to treat (i.e., Wang et al., 2012). The risk of bias for selective reporting was low because all the included trials reported the outcomes as specified in their objectives and methods. The researchers did not detect other potential sources of bias.

### Effect of intervention

#### Primary outcomes

##### HbA1c level.

**Figure 3:**
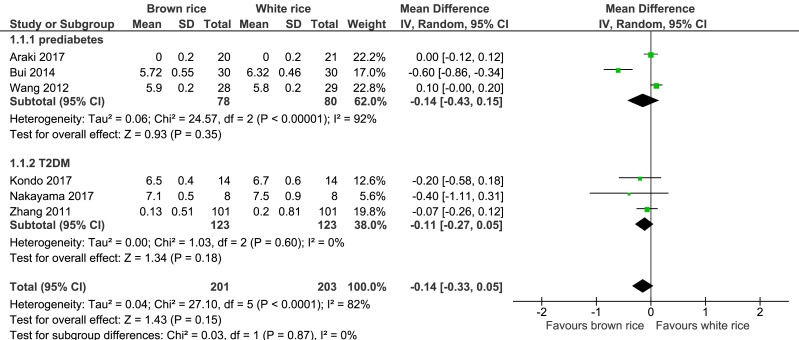
Forest plot of comparison of brown rice and white rice on HbA1c.

Six trials reported on the HbA1c levels of 404 participants. The brown-rice diet did not show any difference in the HbA1c levels compared to the white-rice diet (Fig. 3, Table 2). In view of the considerable heterogeneity, subgroup analysis based on the diagnosis of prediabetes or type 2 diabetes was performed. The results did not reveal any difference between the two groups.

##### Fasting blood glucose (FBG) level.

**Figure 4:**
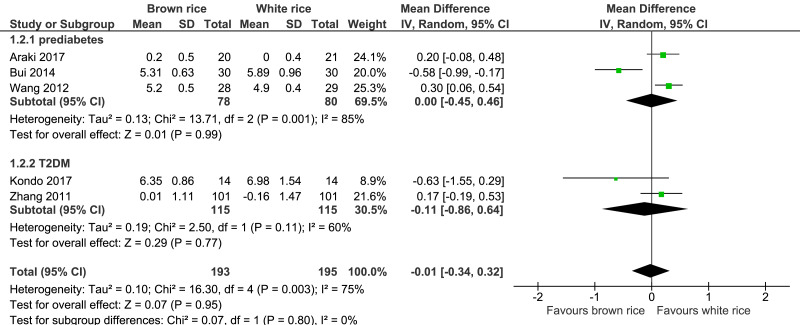
Forest plot of comparison of brown rice and white rice on fasting blood glucose (FBG) level.

Five trials reported on the FBG levels of 388 participants. The brown-rice diet did not show any effect on the FBG level compared to the white-rice diet (Fig. 4, Table 2). Subgroup analysis based on the diagnosis of prediabetes or type 2 diabetes was performed but no difference was found between the two groups.

#### Secondary outcomes

##### Body weight.

**Figure 5:**
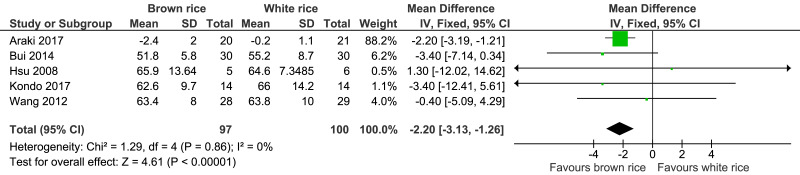
Forest plot of comparison of brown rice and white rice on body weight.

Five trials reported the body weight measurement of 197 participants. The brown-rice-diet group showed a significant body weight reduction compared to the white-rice- diet group, with no significant heterogeneity and with moderate-certainty evidence (Fig. 5, Table 2). In a sensitivity analysis excluding the data from Araki et al. (2017), which contributed 88.2% of the weight, the resulting effect estimate was not significant (*p* = 0.11; MD −2.19; 95% CI [−4.92–0.53]; I2 = 0%).

##### Waist circumference.

**Figure 6:**
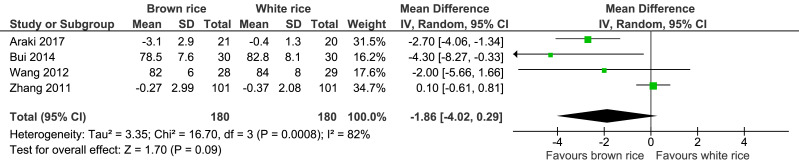
Forest plot of comparison of brown rice and white rice on waist circumference.

Four trials reported the waist circumference measurement of 360 participants. There was no significant difference between the brown-rice and white-rice diet groups, with considerable heterogeneity (I2 = 82%) and moderate-certainty evidence (Fig. 6, Table 2).

##### Systolic blood pressure level.

**Figure 7:**
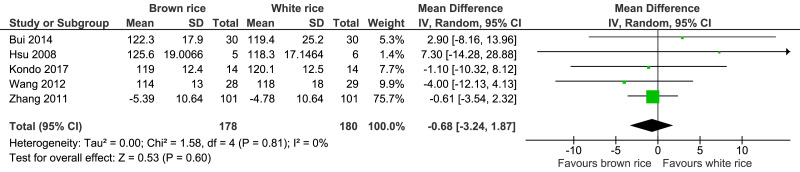
Forest plot of comparison of brown rice and white rice on systolic blood pressure.

Five trials reported the systolic blood pressure levels post-intervention of 358 participants. There was no significant difference in systolic blood pressure level post-intervention between the brown- and white-rice-diet groups. No important heterogeneity was observed (I2 = 0%), and the evidence was of moderate quality (Fig. 7, Table 2).

##### Diastolic blood pressure level.

**Figure 8:**
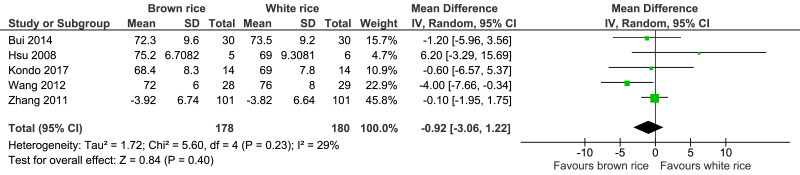
Forest plot of comparison of brown rice and white rice on diastolic blood pressure.

Five trials reported the diastolic blood pressure levels of 358 participants. There was no significant difference in diastolic blood pressure level between the brown and white-rice-diet groups (Fig. 8, Table 2).

##### HDL-cholesterol level.

**Figure 9:**
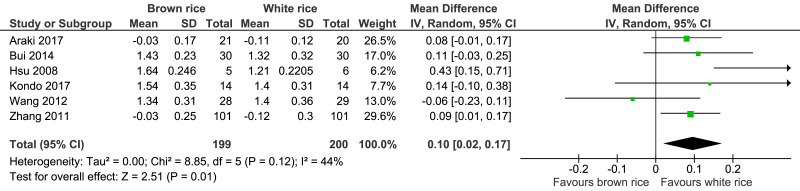
Forest plot of comparison of brown rice and white rice on HDL-cholesterol.

Six trials reported the HDL-cholesterol levels of 399 participants. A significant difference was found between the brown- and white-rice-diet groups. Consuming brown rice increased the HDL-cholesterol level by 0.10 mmol/L (Fig. 9, Table 2).

##### LDL-cholesterol level.

**Figure 10:**
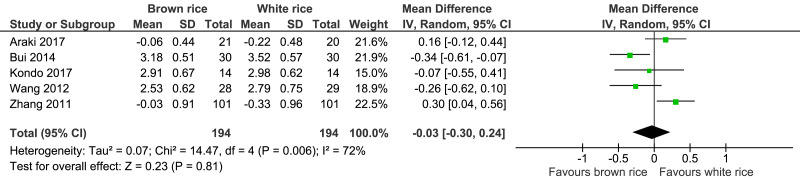
Forest plot of comparison of brown rice and white rice on LDL-cholesterol.

Five trials reported the LDL-cholesterol levels of 388 participants. There was no significant difference between the brown- and white-rice-diet groups in LDL-cholesterol level reduction (Fig. 10, Table 2).

### Sensitivity analysis

After the trials with high and unclear risks of bias for allocation concealment and random sequence generation were removed, there were no substantial changes in the effect sizes or confidence interval (CI) of the outcomes.

### Quality assessment

The researchers in this study used the GRADE approach to evaluate the quality of evidence in the systematic reviews and the strength of the recommendations. The GRADEpro guideline development tool (2015, McMaster University and Evidence Prime, Inc.) was used to grade the quality of evidence for each outcome, and the assessment results are summarised in Table 2. The researchers found that the quality of evidence in the included trials for the primary and secondary outcomes was of low to moderate certainty.

**Table 2:** Summary of findings table.

Outcomes	Anticipated absolute effects^*^(95% CI)		Relative effect (95% CI)	No. of participants (studies)	Certainty of the evidence (GRADE)	Comments
	**Risk with white rice**	**Risk with Brown rice**				
**Brown rice compared to white rice for glycaemic control in prediabetes and type 2 Diabetes Mellitus**						
**Patient or population**: glycaemic control in prediabetes and type 2 Diabetes Mellitus						
**Intervention**: Brown rice						
**Comparison**: white rice						
HbA1c	The mean hbA1c was **0** %	MD **0.14% lower** (0.33 lower to 0.05 higher)	–	404 (6 RCTs)	⊕⊕○○ LOW^a^	
Fasting blood glucose	The mean fasting blood glucose was **0** mmol/L	MD **0.01 mmol/L lower** (0.34 lower to 0.32 higher)	–	388 (5 RCTs)	⊕⊕⊕○ MODERATE^a^	
Body weight	The mean body weight was **0** kg	MD **2.22 kg lower** (3.13 lower to 1.26 lower)	–	197 (5 RCTs)	⊕⊕⊕○ MODERATE^b^	
Waist circumference	The mean waist circumference was **0** cm	MD **1.86 cm lower** (4.02 lower to 0.29 higher)	–	360 (4 RCTs)	⊕⊕○○ LOW^a^^,^^b^	
Systolic blood pressure	The mean systolic blood pressure was **0** mmHg	MD **0.68 mmHg lower** (3.24 lower to 1.87 higher)	–	358 (5 RCTs)	⊕⊕⊕○ MODERATE^b^	
Diastolic blood pressure	The mean diastolic blood pressure was **0** mmHg	MD **0.92 mmHg lower** (3.06 lower to 1.22 higher)	–	358 (5 RCTs)	⊕⊕⊕○ MODERATE^b^	
LDL	The mean LDL was **0** mmol/L	MD **0.03 mmol/L lower** (0.3 higher to 0.24 higher)	–	388 (5 RCTs)	⊕⊕○○ LOW^a^^,^^b^	
HDL	The mean HDL was **0** mmol/L	MD **0.1 mmol/L higher** (0.02 higher to 0.17 higher)	–	399 (6 RCTs)	⊕⊕⊕○ MODERATE^b^	

**Notes.**

* The risk in the intervention group (and its 95% confidence interval) is based on the assumed risk in the comparison group and the **relative effect** of the intervention (and its 95% CI).

CI Confidence interval MD Mean difference

GRADE Working Group grades of evidence.

High certainty: We are very confident that the true effect lies close to that of the estimate of the effect.

Moderate certainty: We are moderately confident in the effect estimate: The true effect is likely to be close to the estimate of the effect, but there is a possibility that it is substantially different.

Low certainty: Our confidence in the effect estimate is limited: The true effect may be substantially different from the estimate of the effect.

Very low certainty: We have very little confidence in the effect estimate: The true effect is likely to be substantially different from the estimate of effect.

a Considerable heterogeneity.

b Small sample size.

### Publication bias

A funnel plot was not drawn due to the small number of included studies.

## Discussion

This meta-analysis reports the effectiveness of a brown-rice diet compared to white rice for glycemic control and metabolic parameters in adults with prediabetes or type 2 diabetes. A total of seven trials involving 415 participants reported the outcomes specified in the objective. Compared with the white-rice-diet group, the brown-rice-diet group did not show any significant improvement in glycemic control in terms of reduction of the HbA1c and FBG levels. Brown rice has a lower glycemic index, which generates a lower postprandial glucose response (Panlasigui & Thompson, 2006). This is supported by a systematic review by Foster-Powell, Holt & Brand-Miller (2002), which found that white rice has a mean glycemic index of 64 and that brown rice has 55. However, the systematic review by Boers, Seijen Ten Hoorn & Mela (2015) on the influence of the characteristics and processing method of rice towards glycemic response leaves doubt as to whether brown rice consistently differs from white rice because longer cooking times are generally required for the preparation of brown rice. The inconsistent difference observed between brown and white rice in this regard supported the absence of significant effects on the HbA1c or FBG levels from this review.

However, brown rice was noted to significantly decreased the body weight by 2.2 kg, but this finding should be cautiously interpreted because only a single trial by Araki et al. (2017) contributed 88.2% of the weight in the meta-analysis. A systematic review and meta-analysis of the cohort studies conducted by Aune et al. (2013) showed that whole-grain intake has a protective association with type 2 diabetes risk by decreasing the energy intake, preventing weight gain and increasing insulin sensitivity. This is consistent with the result of our review that a brown-rice diet significantly reduced the body weight compared with the white-rice diet. Our review also found that after the intake of brown rice as a staple meal, no reduction in waist circumference and blood pressure level were reported. As for the serum lipid profile, the brown-rice diet increased the level of HDL-cholesterol (which is cardioprotective) by 0.10 mmol/L but did not significantly reduce the LDL-cholesterol level in the prediabetes and type 2 diabetes patients.

This study has several strengths. The researchers selected only RCTs in this meta-analysis, which ensured a relatively high-quality study. The evidence provided applies to adult males and females diagnosed with prediabetes or type 2 diabetes. The data obtained were sufficient for analyzing both the primary outcomes (HbA1c and FBG levels) and the secondary outcomes (body weight, waist circumference, systolic blood pressure level, diastolic blood pressure level, LDL-cholesterol level, and HDL-cholesterol level).

This study, however, also has limitations that must be considered. The researchers made conservative judgments with regards to the risk of bias. Six of the seven included trials, with the exception of Kondo et al. (2017), had a high risk of bias in at least two domains (selection bias and performance bias), giventhe obvious differences in appearance and texture between brown and white rice. Five studies were assessed as having an unclear risk of bias in random sequence generation because of the lack of information on the implementation of randomization (Bui et al., 2014; Hsu et al., 2008; Nakayama et al., 2017; Wang et al., 2012; Zhang et al., 2011). The blinding of the outcome assessors was not mentioned in most trials. However, the researchers concluded that the risk of bias was nonetheless low because the outcomes were objectively assessed based on laboratory results and anthropometric measurements. There appeared to be a high risk of attrition bias in view of the incomplete reporting in a trial conducted by Wang et al. (2012). Furthermore, the small sample size in each trial was reflected in the large confidence intervals of the effect estimates. The search strategies used were believed to be comprehensive, but there is still a possibility that some studies were missed.

Another limitation of this study is that the rice samples were not controlled. Commercially available white rice was taken at random rather than being milled from the same batch of brown rice. Three trials did not report the type of brown rice that was used (i.e., Kondo et al., 2017; Wang et al., 2012; Zhang et al., 2011), two trials used pre-germinated brown rice (i.e., Bui et al., 2014; Hsu et al., 2008), one trial used glutinous brown rice (i.e., Nakayama et al., 2017), and another trial used partially abraded brown rice (i.e., Araki et al., 2017). Hence, the variety and physicochemical properties of the rice samples may differ.

The trials that were included in this meta-analysis had a relatively short follow-up duration, ranging from 6 to 16 weeks. In the evaluation of diabetes progression, the HbA1c changes need to be monitored for at least 3 months. Of the six included trials that measured the HbA1c level outcome, four (i.e., Araki et al., 2017; Bui et al., 2014; Wang et al., 2012; Zhang et al., 2011) had an adequate follow-up duration (more than 12 weeks). Two trials had a follow-up duration of less than 12 weeks (i.e., Kondo et al., 2017; Nakayama et al., 2017). HbA1c level is an important indicator of glycemic control. Therefore, more RCTs with larger subjects and a longer follow-up duration are needed to confirm the relationship between a brown-rice diet and glycemic control and metabolic parameters in adults with prediabetes or type 2 diabetes. Additionally, the population size of these trials, which ranged from 28 to 202 participants was indeed limited. Therefore, this meta-analysis may have been underpowered to detect a true effect.

Using the GRADE approach, this review found that the evidence in the included trials for the primary and secondary outcomes were of low to moderate certainty. This is due to the considerable heterogeneity and small sample size. To address the considerable heterogeneity, random-effects model meta-analysis and subgroup analysis were undertaken. However, these analyses generated similar effect estimates and CIs. Further high-quality research is likely to have an important impact on our confidence in the effect estimates and may change such estimates.

## Conclusions

The available evidence indicates that a brown-rice diet in place of a white-rice diet does not significantly affect glycemic control (HbA1c and FBG levels) in prediabetes and type 2 diabetes patients. Brown rice, however, may be used as an alternative for staple foods like refined grains or flour among the aforementioned patients as it has been found to reduce the body weight and to increase the HDL-cholesterol level. The benefits of a brown-rice diet on glycemic control may not be detected in short-term studies. The evidence in this meta-analysis ranged from low to moderate quality. Thus, more high-quality trials with a larger sample size and a longer follow-up duration are needed to further investigate the effects of a brown-rice diet on diabetes glycemic control and metabolic parameters with stronger evidence.

##  Supplemental Information

10.7717/peerj.11291/supp-1 Supplemental Information 1 PRISMA checklist

10.7717/peerj.11291/supp-2 Supplemental Information 2 Rationale and contribution

10.7717/peerj.11291/supp-3 Supplemental Information 3 Search syntax

10.7717/peerj.11291/supp-4 Supplemental Information 4 Data collection form Araki 2017

10.7717/peerj.11291/supp-5 Supplemental Information 5 Data collection form Bui 2014

10.7717/peerj.11291/supp-6 Supplemental Information 6 Data collection form Kondo 2017

10.7717/peerj.11291/supp-7 Supplemental Information 7 Data collection form Wang 2013

10.7717/peerj.11291/supp-8 Supplemental Information 8 Data collection form Zhang 2011

10.7717/peerj.11291/supp-9 Supplemental Information 9 Data collection form Nakayama 2017

10.7717/peerj.11291/supp-10 Supplemental Information 10 Data collection form Hsu 2008

## Additional Information and Declarations

### Data Availability

The following information was supplied regarding data availability: The data extraction forms (raw data) are available in the Supplemental Files.
