# Supplementary material for: The effect of a brown-rice diets on glycemic control and metabolic parameters in prediabetes and type 2 diabetes mellitus: a meta-analysis of randomized controlled trials and controlled clinical trials
Source: PeerJ. 2021 May 26;9:e11291. doi: 10.7717/peerj.11291 (PMC8164413; doi:10.7717/peerj.11291)
Supplement: Supplemental Information 2 [file peerj-09-11291-s002.docx]

Rationale: Current healthcare practitioners recommend consuming brown rice rather than white rice. This is because brown rice is more nutritious and has a lower glycaemic index. The evidence so far suggests that there may be enormous benefits for people with diabetes in rice-consuming populations if white rice is replaced with brown rice. However, the results provided are inconsistent. The purpose of this study is to determine the effectiveness of brown rice on improving glycaemic control and metabolic parameters in prediabetes and type 2 DM.

Contribution: Rice is the staple food for more than half of the global population. White rice is the most commonly consumed type, but brown rice is recognized as a healthier option to maintain good glycaemic control among a rice-consuming population (Imam et al. 2012). Brown rice is a whole grain, while white rice is a refined grain. Studies have shown that white rice, which has a high glycaemic index, increases the risk of diabetes and may worsen the glycaemic control in diabetic patients (Boers et al. 2015). Brown rice is digested slower and results in lower blood glucose response in both healthy and diabetic subjects compared to white rice (Panlasigui & Thompson 2006). In a meta-analysis and systematic review, Asians who consumed white rice had higher risk of developing type 2 diabetes (Hu et al. 2012). In contrast, studies have shown that whole grain intake has a protective effect on type 2 diabetes risk by decreasing the energy intake, preventing weight gain, and increasing insulin sensitivity (Aune et al. 2013; Murtaugh et al. 2003; Parker et al. 2013). A meta-analysis reported that larger whole-grain intake is associated with lower risk of type 2 DM, weight gain, and cardiovascular disease (Ye et al. 2012).
