# Supplementary material for: The effect of a brown-rice diets on glycemic control and metabolic parameters in prediabetes and type 2 diabetes mellitus: a meta-analysis of randomized controlled trials and controlled clinical trials
Source: PeerJ. 2021 May 26;9:e11291. doi: 10.7717/peerj.11291 (PMC8164413; doi:10.7717/peerj.11291)

1. CENTRAL:

ID Search

#1 (brown rice):ti,ab,kw (Word variations have been searched)

#2 (prediabetes):ti,ab,kw (Word variations have been searched)

#3 (diabetes):ti,ab,kw (Word variations have been searched)

#4 (#1 ) AND (#2 OR #3)

#5 (brown rice*):ti,ab,kw AND (diabet* OR prediabet*):ti,ab,kw (Word variations have been searched)


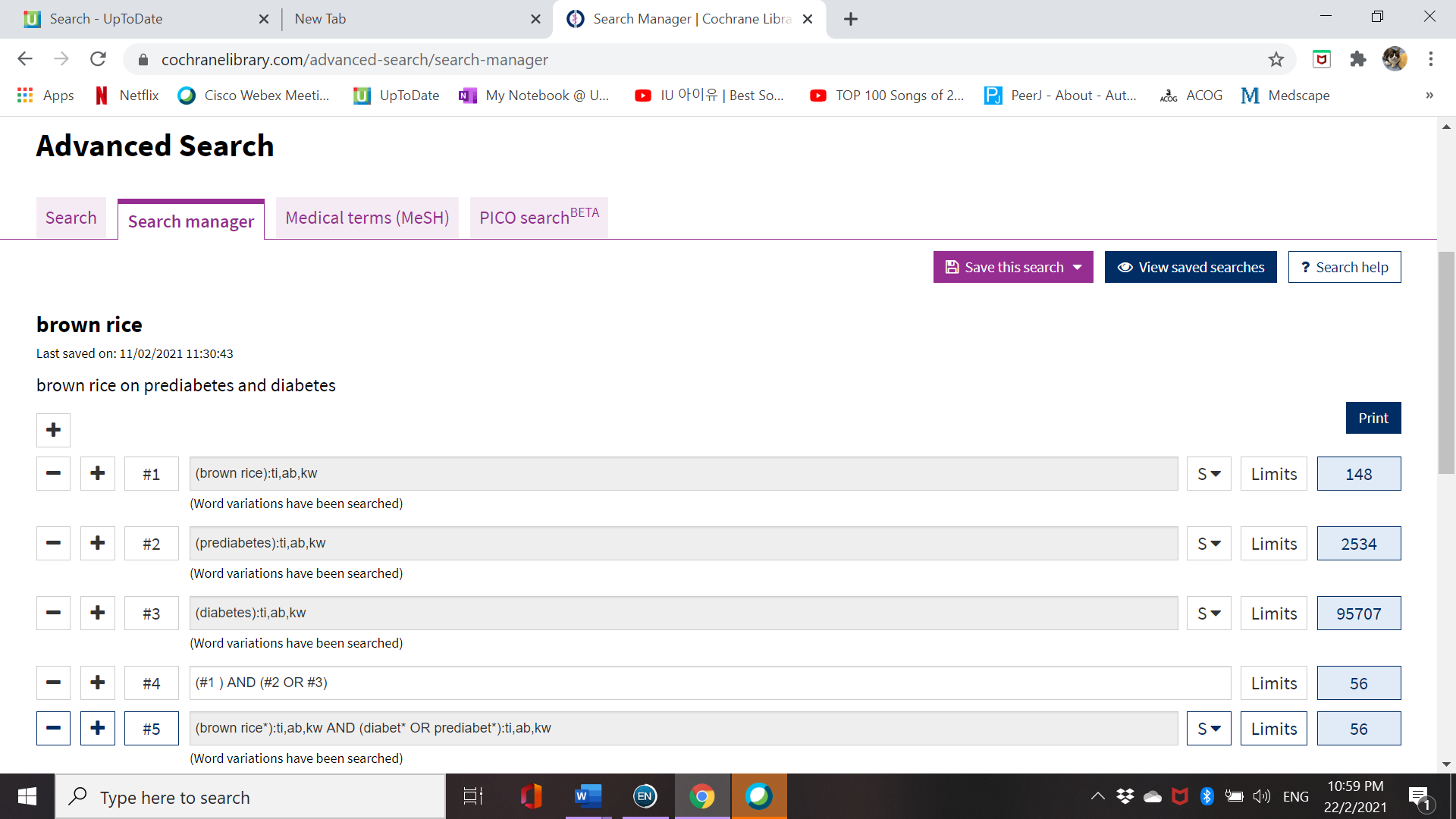


1. Pubmed:

Search: **(brown rice*) AND (diabet* OR prediabet*)**

("brown"[All Fields] OR "browned"[All Fields] OR "browning"[All Fields] OR

"browns"[All Fields]) AND "rice*"[All Fields] AND ("diabet*"[All Fields] OR

"prediabet*"[All Fields])


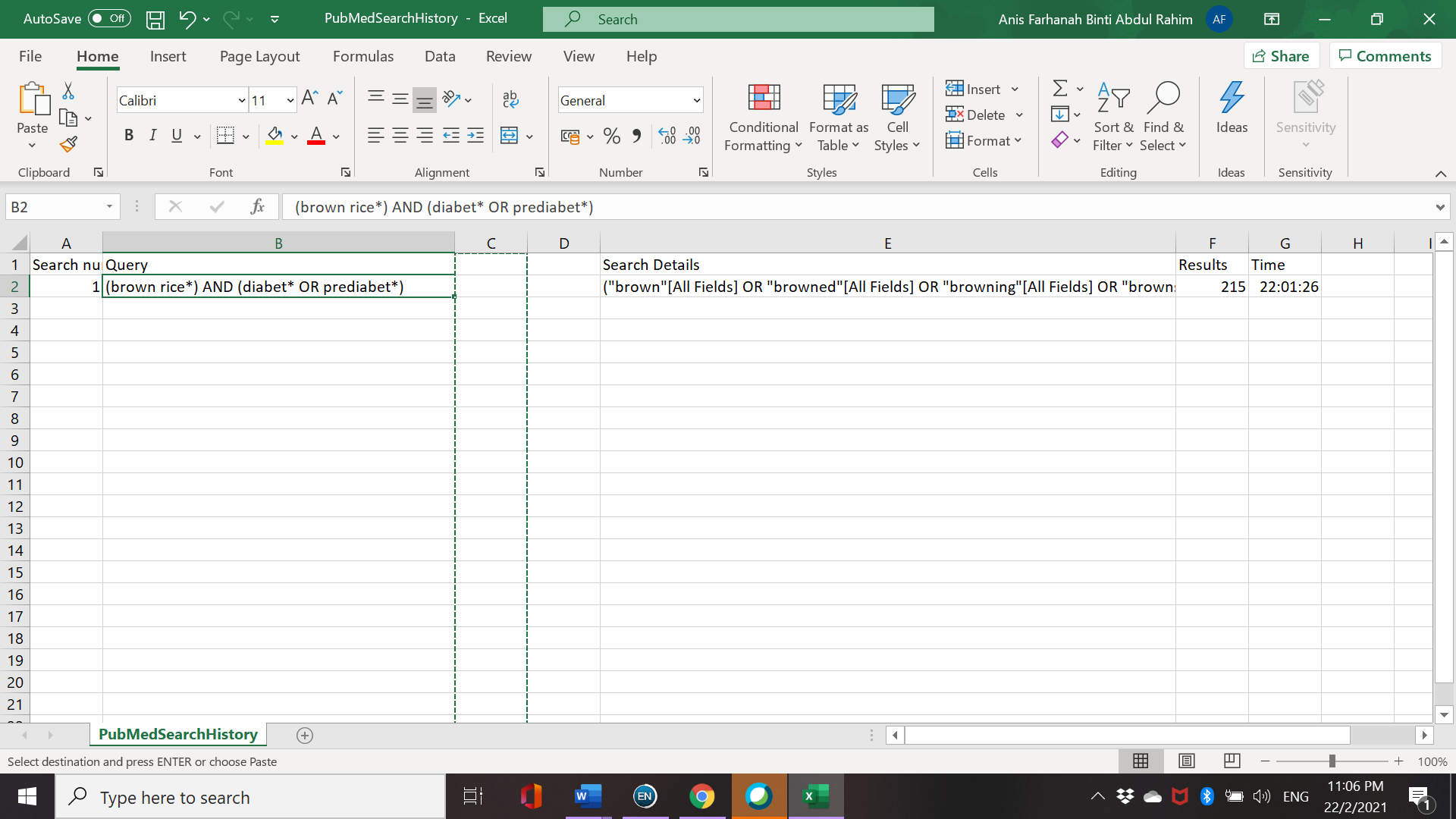

Supplement: Supplemental Information 3 [file peerj-09-11291-s003.docx]
