# Supplementary material for: The effect of a brown-rice diets on glycemic control and metabolic parameters in prediabetes and type 2 diabetes mellitus: a meta-analysis of randomized controlled trials and controlled clinical trials
Source: PeerJ. 2021 May 26;9:e11291. doi: 10.7717/peerj.11291 (PMC8164413; doi:10.7717/peerj.11291)
Supplement: Supplemental Information 4 [file peerj-09-11291-s004.doc]

***Study Eligibility & Data Collection Form***

***General Information***

| **Study ID**  *(e.g. author name, year)* | Araki 2017 |
| --- | --- |
| **Form completed by** | Anis Farhanah binti Abdul Rahim |
| **Study author contact details** | anisfar89@gmail.com |
| **Publication type**  *(e.g. full report, abstract, letter)* | Full report |
| **List of included publications** |  |
| **References of similar trial*** |  |

*This is when the authors published the same study in several reports. All these references to a similar trial should be linked under one *Study ID* in RevMan.

***Study eligibility***

|  | Yes | No | Unclear | Further details |
| --- | --- | --- | --- | --- |
| **RCT/Quasi/CCT** | ***/*** |  |  |  |
| **Relevant participants** | ***/*** |  |  |  |
| **Relevant interventions** | ***/*** |  |  |  |
| **Relevant outcomes*** | ***/*** |  |  |  |

*Include only if the presence of outcomes form the inclusion criterion

If the above answers are ‘YES’, proceed to Section 1.

If any of the above answers are ‘NO*’, record below the information for ‘Excluded studies’

| Reason(s) for exclusion |
| --- |
|  |

Section 1. Characteristics of included studies

This section is to be completed by only one reviewer. State initials: AFAR

| **METHODS** | **Descriptions as stated in paper** |
| --- | --- |
| **Aim of study** *(e.g. efficacy, equivalence, pragmatic)* | To study the effect of continuous intake of partially-abraded brown rice compared to white rice on body weight and indicators of glucose and lipid metabolism in overweight participants with pre-diabetes |
| **Design** *(e.g. parallel, crossover, cluster)* | Parallel group randomized controlled trial |
| **Unit of allocation**  *(by individuals, cluster/ groups or body parts)* |  |
| **Start & end dates** | February 7 to July 2, 2015 |
| **Total study duration** | 12 weeks |
| **Sources of funding**  *(including role of funders)* | The Research Project on Development of Agricultural Products and Foods with Health-promoting benefits. |
| **Possible conflicts of interest**  *(for study authors)* | All authors have declared no conflicts of interest. |

| **PARTICIPANTS** | **Description**  *(include information for each intervention or comparison group)* |
| --- | --- |
| **Population description**  *(Company/companies; occupation)* |  |
| **Setting**  *(including location (city, state, country) and single centre / multicenter)* | University of Tsubaka, Ibaraki, Japan |
| **Inclusion criteria** | 40-64 years old, body mass index (BMI) of 25.0-29.9 kg/m2, and pre-diabetes status (fasting plasma glucose level of 100-125 mg/dL and/or HbA1c level of 5.7%-6.4%). |
| **Exclusion criteria** | Subjects who had received pharmacotherapy for diabetes, dyslipidemia and/or alimentary disease, who participated in other interventional studies, or were current smokers |
| **Method of recruitment of participants** *(e.g. phone, mail, clinic patients, voluntary)* | Participants were recruited via posters in the university and the hospital, or by advertisement in local newspapers. |
| **Total no. randomised** | 42 participants |
| **Clusters**  *(if applicable, no., type, no. people per cluster)* |  |
| **No. randomised per group**  *(specify whether no. people or clusters)* | Intervention: n=21, 1 withdrew consent before the intervention  Control: n=21 |
| **No. missing**  *(if overall, e.g. exclusions & withdrawals, whether or not missing from analysis)* | Intervention: n=1  Control: n=3 |
| **Reasons missing** | Intervention: n=1 for personal reasons  Control: n=3, 2 for personal reason, 1 for health problem |
| **Baseline imbalances** | Balanced |
| **Age** | WR: 52.9±2.5 years  PABR: 54.8±2.2 years |
| **Sex (proportion)** | WR: 57.1% female  PABR: 50% female |
| **Race/Ethnicity** | Japanese |
| **Other relevant sociodemographics** |  |
| **Subgroups measured** *(eg split by age or sex)* |  |
| **Subgroups reported** | Subgroup analyses performed with 22 participants (10 from WR group and 12 from PABR group) |

Section 2. Risk of bias assessment

We recommend you refer to and use the method described in the Cochrane Handbook.

This section is completed by two reviewers. State initials: (i) AFAR (ii) NMN

| **Domain** | **Risk of bias** | | | **Support for judgement**  *(include direct quotes where available with explanatory comments)* | **Location in text or source** *(page, table)* |
| --- | --- | --- | --- | --- | --- |
|  | Low | High | Unclear |  |  |
| **Random sequence generation**  *(selection bias)* | Low |  |  | Quote: “The participants were allocated to receive either PABR or WR with an allocation table prepared by a data coordinator based on simple randomization method with stratification by sex and low-density lipoprotein cholesterol (LDL-C) levels (>140 mg/dL or not).” | Page e2 |
| **Allocation concealment**  *(selection bias)* |  | High |  | Quote: “each participant received an electric rice-cooker and un-cooked rice. Participants were required to cook test meals by themselves with the instructed method. When cooking, the rice: water ratios were 1:1 and 1:2 in weight for WR and PABR, respectively. Both types of rice were steamed using the cooking mode for WR” | Page e2 |
| **Blinding of participants and personnel**  *(performance bias)* |  | High |  | Quote: “each participant received an electric rice-cooker and un-cooked rice. Participants were required to cook test meals by themselves with the instructed method. When cooking, the rice: water ratios were 1:1 and 1:2 in weight for WR and PABR, respectively. Both types of rice were steamed using the cooking mode for WR” | Page e2 |
| **Blinding of outcome assessment**  *(detection bias)* |  |  | Unclear | Comment: all the anthropometric measurement and biochemical parameters were done in laboratory but does not mentioned whether the technicians were blinded or not | Page e3 |
| **Incomplete outcome data**  *(attrition bias)* |  | High |  | Comment: total number of participants completed the intervention n=18 for WR, n=19 for PABR, however | Figure 1, page e3 |
| **Selective outcome reporting**  *(reporting bias)* |  | High |  | Quote: “log-transformations were applied for serum TG levels” | Page e4 |
| **Other bias** |  | High |  | Comment: n=6 in WR group, n=8 in PABR group has discontinuation period which will effect the end result | Figure 1, page e3 |

Random sequence generation = Process used to assign people into intervention and control groups

Allocation concealment = Process used to prevent foreknowledge of group assignment in a RCT

Blinding of participants and personnel = Presence or absence of blinding for participants and health personnel

Blinding of outcome assessment = presence or absence of blinding for assessment of outcome

Incomplete outcome data = application of intention-to-treat analysis is one in which all the participants in a trial are analysed according to the intervention to which they were allocated

Selective outcome reporting = Selection of a subset of the original variables recorded

***Section 3. Intervention groups***

This section is completed by two reviewers. State initials: (i) AFAR (ii) NMN

| **Outcomes relevant to your review**  *(Copy and paste from ‘Types of outcome measures’)* | **Reported in paper**  *(Yes / No)* | **Outcome definition** *(with diagnostic criteria if relevant)* | **Unit of measurement & tool**  *(if relevant)* | **Reanalysis required?** *(specify)* |
| --- | --- | --- | --- | --- |
| HbA1c | Yes |  | % |  |
| FPG | Yes |  | mg/dL |  |
| Body weight | Yes |  | Kg |  |
| Waist circumference | Yes |  | Cm |  |
| Blood pressure | No |  | - |  |
| LDL-cholesterol | Yes |  | mg/dL |  |
| HDL-cholesterol | Yes |  | mg/dL |  |

***Section 4. Data and analysis***

| **DICHOTOMOUS OUTCOME** | Intervention group | | Control group | |
| --- | --- | --- | --- | --- |
|  | Number of events | Number of participants | Number of events | Number of participants |
|  |  |  |  |  |
|  |  |  |  |  |
|  |  |  |  |  |
|  |  |  |  |  |
|  |  |  |  |  |
|  |  |  |  |  |

State details if outcomes were only described in text or figures.

| **CONTINUOUS OUTCOME** | Unit of measurement | Intervention group | | Control group | |
| --- | --- | --- | --- | --- | --- |
|  |  | n | Mean (SD) | n | Mean (SD) |
| HbA1c | % | 21 | -0.1 (0.1) | 20 | -0.1 (0.1) |
| FPG | mg/dL | 21 | 1.8 (4.6) | 20 | 0.4 (6.2) |
| Body weight | Kg | 21 | -2.4 (2.0) | 20 | -0.2 (1.1) |
| Waist circumference | Cm | 21 | -3.1 (2.9) | 20 | -0.4 (1.3) |
| Blood pressure | - | - | - | - | - |
| LDL-cholesterol | mg/dL | 21 | -8.4 (13.0) | 20 | -6.7 (15.1) |
| HDL-cholesterol | mg/dL | 21 | -2.2 (6.5) | 20 | -2.2 (7.0) |

State details if outcomes were only described in text or figures.

***Section 5. Other information***

|  | **Description as stated in paper** |
| --- | --- |
| **Key conclusions of study authors** | In conclusion, the intake of 200-g PABR twice a day for 12 weeks was considered beneficial for body weight loss and lipid metabolism improvement in overweight participants with pre-diabetes, and these results might have been incurred through the influence of insoluble dietary fiber. |
| **Results that you calculated using a formula** |  |
| **References to other relevant studies**  *(Did this report include any references to unpublished data from potentially eligible trials not already identified for this review? If yes, give list contact name and details)* |  |
| **Correspondence required for further study information** *(from whom, what and when)* |  |

**Sources:**

Higgins JPT, Green S (editors). Cochrane Handbook for Systematic Reviews of Interventions Version 5.1.0 [updated March 2011]. The Cochrane Collaboration, 2011.Available from www.cochrane-handbook.org.
