# Supplementary material for: The effect of a brown-rice diets on glycemic control and metabolic parameters in prediabetes and type 2 diabetes mellitus: a meta-analysis of randomized controlled trials and controlled clinical trials
Source: PeerJ. 2021 May 26;9:e11291. doi: 10.7717/peerj.11291 (PMC8164413; doi:10.7717/peerj.11291)
Supplement: Supplemental Information 5 [file peerj-09-11291-s005.doc]

***Study Eligibility & Data Collection Form***

***General Information***

| **Study ID**  *(e.g. author name, year)* | Bui, 2014 |
| --- | --- |
| **Form completed by** | Anis Farhanah binti Abdul Rahim |
| **Study author contact details** | anisfar89@gmail.com |
| **Publication type**  *(e.g. full report, abstract, letter)* |  |
| **List of included publications** |  |
| **References of similar trial*** |  |

*This is when the authors published the same study in several reports. All these references to a similar trial should be linked under one *Study ID* in RevMan.

***Study eligibility***

|  | Yes | No | Unclear | Further details |
| --- | --- | --- | --- | --- |
| **RCT/Quasi/CCT** | ***/*** |  |  |  |
| **Relevant participants** | ***/*** |  |  |  |
| **Relevant interventions** | ***/*** |  |  |  |
| **Relevant outcomes*** | ***/*** |  |  |  |

*Include only if the presence of outcomes form the inclusion criterion

If the above answers are ‘YES’, proceed to Section 1.

If any of the above answers are ‘NO*’, record below the information for ‘Excluded studies’

| Reason(s) for exclusion |
| --- |
|  |

Section 1. Characteristics of included studies

This section is to be completed by only one reviewer. State initials: AFAR

| **METHODS** | **Descriptions as stated in paper** |
| --- | --- |
| **Aim of study** *(e.g. efficacy, equivalence, pragmatic)* | To evaluate the effect of pre-germinated brown rice administration for 16 weeks on various parameters in Vietnamese women. |
| **Design** *(e.g. parallel, crossover, cluster)* | Controlled clinical trial |
| **Unit of allocation**  *(by individuals, cluster/ groups or body parts)* |  |
| **Start & end dates** | 2011 |
| **Total study duration** | 16 weeks |
| **Sources of funding**  *(including role of funders)* | Vietnam’s National Foundation for Science and Technology Department (NAFOSTED), grant no 106.99.140.09 from the Ministry of Science and Technology, Vietnam. |
| **Possible conflicts of interest**  *(for study authors)* | Not stated |

| **PARTICIPANTS** | **Description**  *(include information for each intervention or comparison group)* |
| --- | --- |
| **Population description**  *(Company/companies; occupation)* |  |
| **Setting**  *(including location (city, state, country) and single centre / multicenter)* | Hai Duong Province, located in the Red River Delta 57km from Hanoi. |
| **Inclusion criteria** | Women aged 45 to 65 years  IGT defined by a 2-hour post 50 g glucose load, a blood glucose level of 7.8 to 11 mmol/L. |
| **Exclusion criteria** | DM patient on medication |
| **Method of recruitment of participants** *(e.g. phone, mail, clinic patients, voluntary)* | Screening |
| **Total no. randomised** | No randomisation |
| **Clusters**  *(if applicable, no., type, no. people per cluster)* |  |
| **No. randomised per group**  *(specify whether no. people or clusters)* | Intervention: n=30  Control: n=30 |
| **No. missing**  *(if overall, e.g. exclusions & withdrawals, whether or not missing from analysis)* | Intervention: nil  Control: nil |
| **Reasons missing** | Intervention: nil  Control: nil |
| **Baseline imbalances** | Balanced |
| **Age** | 56.9(5.8) in PGBR group  56.6(5.0) in WR group |
| **Sex (proportion)** | Women only |
| **Race/Ethnicity** | Vietnamese |
| **Other relevant sociodemographics** |  |
| **Subgroups measured** *(eg split by age or sex)* |  |
| **Subgroups reported** |  |

Section 2. Risk of bias assessment

We recommend you refer to and use the method described in the Cochrane Handbook.

This section is completed by two reviewers. State initials: (i)AFAR (ii) NMN

| **Domain** | **Risk of bias** | | | **Support for judgement**  *(include direct quotes where available with explanatory comments)* | **Location in text or source** *(page, table)* |
| --- | --- | --- | --- | --- | --- |
|  | Low | High | Unclear |  |  |
| **Random sequence generation**  *(selection bias)* |  | High |  | Comment: this is a controlled clinical trial, thus randomization was not done. |  |
| **Allocation concealment**  *(selection bias)* |  | High |  | Quote: “to help the subjects in the PGBR group to adapt, WR was replaced over the course of a month.” | Page 184 |
| **Blinding of participants and personnel**  *(performance bias)* |  | High |  | Quote: “the potential beneficial health effects of whole grain were explained to all subjects and informed consent was obtained from each participant.” | Page 184 |
| **Blinding of outcome assessment**  *(detection bias)* |  |  | Unclear | Comment: all the anthropometric measurement and biochemical parameters were done in laboratory but does not mentioned whether the technicians were blinded or not | Page 184 |
| **Incomplete outcome data**  *(attrition bias)* | Low |  |  | Comment: all outcomes were reported | Table 2 page 185 |
| **Selective outcome reporting**  *(reporting bias)* | Low |  |  | Comment: all outcomes were analysed | Table 1 page 184 |
| **Other bias** |  | High |  | Comment: this is a clinical controlled trial where participants aware of intervention | Page 184 |

Random sequence generation = Process used to assign people into intervention and control groups

Allocation concealment = Process used to prevent foreknowledge of group assignment in a RCT

Blinding of participants and personnel = Presence or absence of blinding for participants and health personnel

Blinding of outcome assessment = presence or absence of blinding for assessment of outcome

Incomplete outcome data = application of intention-to-treat analysis is one in which all the participants in a trial are analysed according to the intervention to which they were allocated

Selective outcome reporting = Selection of a subset of the original variables recorded

***Section 3. Intervention groups***

This section is completed by two reviewers. State initials: (i)AFAR (ii) NMN

| **Outcomes relevant to your review**  *(Copy and paste from ‘Types of outcome measures’)* | **Reported in paper**  *(Yes / No)* | **Outcome definition** *(with diagnostic criteria if relevant)* | **Unit of measurement & tool**  *(if relevant)* | **Reanalysis required?** *(specify)* |
| --- | --- | --- | --- | --- |
| HbA1c | Yes |  | % |  |
| FBG | Yes |  | mmol/L |  |
| Body weight | Yes |  | kg, |  |
| Waist circumference | Yes |  | cm, |  |
| Blood pressure | Yes |  | mmHg |  |
| LDL-cholesterol | Yes |  | mmol/L |  |
| HDL-cholesterol | Yes |  | mmol/L |  |

***Section 4. Data and analysis***

| **DICHOTOMOUS OUTCOME** | Intervention group | | Control group | |
| --- | --- | --- | --- | --- |
|  | Number of events | Number of participants | Number of events | Number of participants |
|  |  |  |  |  |
|  |  |  |  |  |
|  |  |  |  |  |
|  |  |  |  |  |
|  |  |  |  |  |

State details if outcomes were only described in text or figures.

| **CONTINUOUS OUTCOME** | Unit of measurement | Intervention group | | Control group | |
| --- | --- | --- | --- | --- | --- |
|  |  | n | Mean (SD) | n | Mean (SD) |
| HbA1c | % | 30 | 5.72 (0.55) | 30 | 6.32 (0.46) |
| FBG | mmol/L | 30 | 5.31 (0.63) | 30 | 5.89 (0.96) |
| Body weight | kg | 30 | 51.8 (5.8) | 30 | 55.2 (8.7) |
| Waist circumference | cm | 30 | 78.5 (7.6) | 30 | 82.8 (8.1) |
| Blood pressure | mmHg | 30 | SBP 122.3 (17.9)  DBP 72.3 (9.6) | 30 | SBP 119.4 (25.2)  DBP 73.5 (9.2) |
| LDL-cholesterol | mmol/L | 30 | 3.18 (0.51) | 30 | 3.52 (0.57) |
| HDL-cholesterol | mmol/L | 30 | 1.43 (0.23) | 30 | 1.32 (0.32) |

State details if outcomes were only described in text or figures.

***Section 5. Other information***

|  | **Description as stated in paper** |
| --- | --- |
| **Key conclusions of study authors** | Replacing white rice with pre-germinated brown rice is useful in controlling blood glucose and lipid levels as well as body weight in people who consume rice as a staple food. |
| **Results that you calculated using a formula** |  |
| **References to other relevant studies**  *(Did this report include any references to unpublished data from potentially eligible trials not already identified for this review? If yes, give list contact name and details)* |  |
| **Correspondence required for further study information** *(from whom, what and when)* |  |

**Sources:**

Higgins JPT, Green S (editors). Cochrane Handbook for Systematic Reviews of Interventions Version 5.1.0 [updated March 2011]. The Cochrane Collaboration, 2011.Available from www.cochrane-handbook.org.
