# Supplementary material for: The effect of a brown-rice diets on glycemic control and metabolic parameters in prediabetes and type 2 diabetes mellitus: a meta-analysis of randomized controlled trials and controlled clinical trials
Source: PeerJ. 2021 May 26;9:e11291. doi: 10.7717/peerj.11291 (PMC8164413; doi:10.7717/peerj.11291)
Supplement: Supplemental Information 6 [file peerj-09-11291-s006.doc]

***Study Eligibility & Data Collection Form***

***General Information***

| **Study ID**  *(e.g. author name, year)* | Kondo, 2017 |
| --- | --- |
| **Form completed by** | Anis Farhanah binti Abdul Rahim |
| **Study author contact details** | anisfar89@gmail.com |
| **Publication type**  *(e.g. full report, abstract, letter)* | Full Report |
| **List of included publications** |  |
| **References of similar trial*** |  |

*This is when the authors published the same study in several reports. All these references to a similar trial should be linked under one *Study ID* in RevMan.

***Study eligibility***

|  | Yes | No | Unclear | Further details |
| --- | --- | --- | --- | --- |
| **RCT/Quasi/CCT** | ***/*** |  |  |  |
| **Relevant participants** | ***/*** |  |  |  |
| **Relevant interventions** | ***/*** |  |  |  |
| **Relevant outcomes*** | ***/*** |  |  |  |

*Include only if the presence of outcomes form the inclusion criterion

If the above answers are ‘YES’, proceed to Section 1.

If any of the above answers are ‘NO*’, record below the information for ‘Excluded studies’

| Reason(s) for exclusion |
| --- |
|  |

Section 1. Characteristics of included studies

This section is to be completed by only one reviewer. State initials: ……

| **METHODS** | **Descriptions as stated in paper** |
| --- | --- |
| **Aim of study** *(e.g. efficacy, equivalence, pragmatic)* | To study the effect of fiber-rich diet with brown rice on endothelial function in patients with type 2 diabetes mellitus. |
| **Design** *(e.g. parallel, crossover, cluster)* | Randomized, open-labeled, parallel-controlled trial |
| **Unit of allocation**  *(by individuals, cluster/ groups or body parts)* |  |
| **Start & end dates** | April 3, 2012 till December 9, 2014 |
| **Total study duration** | 8 weeks |
| **Sources of funding**  *(including role of funders)* | This study was funded by the Department of Medicine, Shiga University of Medical Science (SUMS), and Sunstar Inc. This study was also supported, in part, by a Grant-in-Aid from the Japan Society for the Promotion of Science KAKENHI, grant number 25750346 (to K. Kondo), and the Japan Foundation for Applied Enzymology (to K. Kondo). The Department of Medicine at SUMS received research promotion grants (Shougaku Kifukin) from Ono Pharmaceutical, Takeda, Boehringer Ingelheim, Astellas, AstraZeneca, Sunstar, Taisho-Toyama, Daiichi-Sankyo, and MSD. The research topics of these grants were not restricted. The funders had no role in the study design, data collection and analysis, decision to publish, or preparation of the manuscript except for Sunstar. Atsushi Ishikado, Fumio Nikami, Makoto Suwa, Motonobu Matsumoto and Taketoshi Makino are employed by Sunstar Inc. Sunstar Inc. provided support in the form of salaries for authors AI, F. Nikami, MS, MM and TM, and provided brown rice products but did not have any additional role in the data collection and analysis, and decision to publish. AI contributed to the study design, AI, F. Nikami, MS, MM and TM reviewed and edited final version of the manuscript. The specific roles of these authors are articulated in the ‘author contributions’ section. Fumiyuki Nakagawa is employed by CMIC Pharma Science Co. CMIC Pharma Science Co. provided support in the form of salary for author F. Nakagawa, but did not have role in the study design, data collection and analysis, and decision to publish. F. Nakagawa reviewed and edited final version of the manuscript. The specific role of this author is articulated in the ‘author contributions’ section. |
| **Possible conflicts of interest**  *(for study authors)* | We have the following interests: The Department of Medicine at Shiga University of Medical Science (SUMS) received research promotion grants (Shougaku Kifukin) from Sunstar and this study was partially funded by Sunstar. A. Ishikado is an employee of Sunstar and a visiting assistant professor of SUMS. Fumio Nikami, Makoto Suwa, Motonobu Matsumoto and Taketoshi Makino are also employed by Sunstar Inc. F. Nakagawa is an employee of CMIC Pharma Science Co., and a graduate student of SUMS. The Department of Medicine at SUMS received research promotion grants (Shougaku Kifukin) from Ono Pharmaceutical, Takeda, Boehringer Ingelheim, Astellas, AstraZeneca, Sunstar, Taisho- Toyama, Daiichi-Sankyo, and MSD. The research topics of these grants were not restricted. There are no patents, products in development or marketed products to declare other than brown rice pouch, which is a marketed product of Sunstar Inc. |

| **PARTICIPANTS** | **Description**  *(include information for each intervention or comparison group)* |
| --- | --- |
| **Population description**  *(Company/companies; occupation)* |  |
| **Setting**  *(including location (city, state, country) and single centre / multicenter)* | Shiga University of Medical Science Hospital, Shiga, Japan |
| **Inclusion criteria** | 1. T2DM aged between 40 and 80 years 2. HbA1c < 8.4% |
| **Exclusion criteria** | Individuals taking an α-glucosidase inhibitor, consuming brown rice or dietary fiber supplements, undergoing insulin treatment, who had changed medications for T2DM within the previous 2 months, who were current smokers, who were pregnant or breastfeeding, or who had severe vascular, hepatic, renal, or infectious diseases or cancer |
| **Method of recruitment of participants** *(e.g. phone, mail, clinic patients, voluntary)* | Not stated |
| **Total no. randomised** | 29 patients |
| **Clusters**  *(if applicable, no., type, no. people per cluster)* |  |
| **No. randomised per group**  *(specify whether no. people or clusters)* | Intervention: n=14  Control: n=15 |
| **No. missing**  *(if overall, e.g. exclusions & withdrawals, whether or not missing from analysis)* | Intervention: nil  Control: n=1, consent withdrawal |
| **Reasons missing** | Intervention: nil  Control: consent withdrawal |
| **Baseline imbalances** | Absent |
| **Age** | Brown rice group: 65.2±8.7 years old  White rice group: 68.1±6.8 years old |
| **Sex (proportion)** | 9 male/ 5 female |
| **Race/Ethnicity** | Japanese |
| **Other relevant sociodemographics** |  |
| **Subgroups measured** *(eg split by age or sex)* |  |
| **Subgroups reported** |  |

Section 2. Risk of bias assessment

We recommend you refer to and use the method described in the Cochrane Handbook.

This section is completed by two reviewers. State initials: (i)AFAR (ii) NMN

| **Domain** | **Risk of bias** | | | **Support for judgement**  *(include direct quotes where available with explanatory comments)* | **Location in text or source** *(page, table)* |
| --- | --- | --- | --- | --- | --- |
|  | Low | High | Unclear |  |  |
| **Random sequence generation**  *(selection bias)* | Low |  |  | Quote: “Randomization was stratified by age, sex, and body mass index (BMI). We used the minimization method for randomization. The number of strata for each factor was two. The cut off values for stratification factors were an age of 66 years and a BMI of 24. After the run-in period, participants were randomly assigned to a brown rice or white rice diet.” | Page 5 |
| **Allocation concealment**  *(selection bias)* | Low |  |  | Quote: “Both brown rice (for the fiber-rich diet) and white rice were provided in small single-serve pouches for the 8-week study to limit energy intake (provided by Sunstar Inc., Osaka, Japan). Barley and amaranth were blended with brown rice by the company to adjust the viscosity and flavor.” | Page 5 |
| **Blinding of participants and personnel**  *(performance bias)* | Low |  |  | Quote: “Investigators were provided with a random allocation sequence made by a research assistant, who was independent of the investigators, using computer-generated random digits.” | Page 5 |
| **Blinding of outcome assessment**  *(detection bias)* |  |  | Unclear | Comment: all the anthropometric measurement and biochemical parameters were done in laboratory but does not mentioned whether the technicians were blinded or not | Page 6 |
| **Incomplete outcome data**  *(attrition bias)* | Low |  |  | Comment: only 1 patient loss to follow-up | Figure 1 |
| **Selective outcome reporting**  *(reporting bias)* | Low |  |  | Comment: all outcomes were reported | Page 6,11 |
| **Other bias** |  |  | Unclear |  |  |

Random sequence generation = Process used to assign people into intervention and control groups

Allocation concealment = Process used to prevent foreknowledge of group assignment in a RCT

Blinding of participants and personnel = Presence or absence of blinding for participants and health personnel

Blinding of outcome assessment = presence or absence of blinding for assessment of outcome

Incomplete outcome data = application of intention-to-treat analysis is one in which all the participants in a trial are analysed according to the intervention to which they were allocated

Selective outcome reporting = Selection of a subset of the original variables recorded

***Section 3. Intervention groups***

This section is completed by two reviewers. State initials: (i)AFAR (ii) NMN

| **Outcomes relevant to your review**  *(Copy and paste from ‘Types of outcome measures’)* | **Reported in paper**  *(Yes / No)* | **Outcome definition** *(with diagnostic criteria if relevant)* | **Unit of measurement & tool**  *(if relevant)* | **Reanalysis required?** *(specify)* |
| --- | --- | --- | --- | --- |
| HbA1c | Yes |  | %, latex agglutination method |  |
| FBS | Yes |  | mmol/L, hexokinase glucose 6-phosphate dehydrogenase ultraviolet method |  |
| Body weight | Yes |  | kg, bodyweight of each participant, without shoes and with light clothing weighing a maximum of 0.1 kg, was recorded using an electronic scale |  |
| Waist circumference | No |  | - |  |
| Blood pressure | Yes |  | mmHg |  |
| LDL-cholesterol | Yes |  | mmol/L, calculated using the Friedewald equation (total  cholesterol − [HDL-cholesterol + triglyceride/5]). |  |
| HDL-cholesterol | Yes |  | mmol/L, direct method |  |

***Section 4. Data and analysis***

| **DICHOTOMOUS OUTCOME** | Intervention group | | Control group | |
| --- | --- | --- | --- | --- |
|  | Number of events | Number of participants | Number of events | Number of participants |
|  |  |  |  |  |
|  |  |  |  |  |
|  |  |  |  |  |
|  |  |  |  |  |
|  |  |  |  |  |
|  |  |  |  |  |

State details if outcomes were only described in text or figures.

| **CONTINUOUS OUTCOME** | Unit of measurement | Intervention group | | Control group | |
| --- | --- | --- | --- | --- | --- |
|  |  | n | Mean (SD) | n | Mean (SD) |
| HbA1c | % | 14 | 6.5 (0.4) | 14 | 6.7 (0.6) |
| FBS | mmol/L | 14 | 6.35 (0.86) | 14 | 6.98 (1.54) |
| Body weight | kg | 14 | 62.6 (9.7) | 14 | 66.0 (14.2) |
| Waist circumference | - |  |  |  |  |
| Blood pressure | mmHg | 14 | SBP 119.0 (12.4)  DBP 68.4 (8.3) | 14 | SBP 120.1 (12.5)  DBP 69.0 (7.8) |
| LDL-cholesterol | mmol/L | 14 | 2.91 (0.67) | 14 | 2.98 (0.62) |
| HDL-cholesterol | mmol/L | 14 | 1.54 (0.35) | 14 | 1.40 (0.31) |

State details if outcomes were only described in text or figures.

***Section 5. Other information***

|  | **Description as stated in paper** |
| --- | --- |
| **Key conclusions of study authors** | In conclusion, intervention with a fiber-rich diet with brown rice for 8 weeks effectively improved endothelial function, without changing HbA1c levels, possibly through reducing glucose excursions. |
| **Results that you calculated using a formula** |  |
| **References to other relevant studies**  *(Did this report include any references to unpublished data from potentially eligible trials not already identified for this review? If yes, give list contact name and details)* |  |
| **Correspondence required for further study information** *(from whom, what and when)* |  |

**Sources:**

Higgins JPT, Green S (editors). Cochrane Handbook for Systematic Reviews of Interventions Version 5.1.0 [updated March 2011]. The Cochrane Collaboration, 2011.Available from www.cochrane-handbook.org.
