# Supplementary material for: The effect of a brown-rice diets on glycemic control and metabolic parameters in prediabetes and type 2 diabetes mellitus: a meta-analysis of randomized controlled trials and controlled clinical trials
Source: PeerJ. 2021 May 26;9:e11291. doi: 10.7717/peerj.11291 (PMC8164413; doi:10.7717/peerj.11291)
Supplement: Supplemental Information 7 [file peerj-09-11291-s007.doc]

***Study Eligibility & Data Collection Form***

***General Information***

| **Study ID**  *(e.g. author name, year)* | Wang, 2013 |
| --- | --- |
| **Form completed by** | Anis Farhanah binti Abdul Rahim |
| **Study author contact details** | anisfar89@gmail.com |
| **Publication type**  *(e.g. full report, abstract, letter)* | Full report |
| **List of included publications** |  |
| **References of similar trial*** |  |

*This is when the authors published the same study in several reports. All these references to a similar trial should be linked under one *Study ID* in RevMan.

***Study eligibility***

|  | Yes | No | Unclear | Further details |
| --- | --- | --- | --- | --- |
| **RCT/Quasi/CCT** | **/** |  |  |  |
| **Relevant participants** | **/** |  |  |  |
| **Relevant interventions** | **/** |  |  |  |
| **Relevant outcomes*** | **/** |  |  |  |

*Include only if the presence of outcomes form the inclusion criterion

If the above answers are ‘YES’, proceed to Section 1.

If any of the above answers are ‘NO*’, record below the information for ‘Excluded studies’

| Reason(s) for exclusion |
| --- |
|  |

Section 1. Characteristics of included studies

This section is to be completed by only one reviewer. State initials: AFAR

| **METHODS** | **Descriptions as stated in paper** |
| --- | --- |
| **Aim of study** *(e.g. efficacy, equivalence, pragmatic)* | To test the effect of substituting brown rice for white rice on insulin resistance. |
| **Design** *(e.g. parallel, crossover, cluster)* | Randomized prospective study. |
| **Unit of allocation**  *(by individuals, cluster/ groups or body parts)* |  |
| **Start & end dates** | Not stated |
| **Total study duration** | 12 weeks |
| **Sources of funding**  *(including role of funders)* | None. |
| **Possible conflicts of interest**  *(for study authors)* | None of the authors declare any conflicts of interest. |

| **PARTICIPANTS** | **Description**  *(include information for each intervention or comparison group)* |
| --- | --- |
| **Population description**  *(Company/companies; occupation)* |  |
| **Setting**  *(including location (city, state, country) and single centre / multicenter)* | Outpatient clinic in Flushing, New York City |
| **Inclusion criteria** | Any adult subject with pre-diabetes (defined as hemoglobin A1c levels between 5.7 and 6.4%) |
| **Exclusion criteria** | a) diagnosis of diabetes mellitus, renal disease or cardiovascular disease (apart from hypertension),  b) body weight fluctuation of more than 10% in the past 6 months,  c) planning to change significantly level of physical activity during the time of study,  d) planning to move out of town or take a vacation for ≥ 14 days during the time of the study,  e) current smoker,  g) consumption of greater than 2 alcoholic drinks per day,  h) allergy to any type of grain,  i) special diets,  j) use of medications that would affect blood glucose levels (e.g. steroids) and  g) any current malignancy. |
| **Method of recruitment of participants** *(e.g. phone, mail, clinic patients, voluntary)* | Outpatient clinic |
| **Total no. randomised** | 100 patients |
| **Clusters**  *(if applicable, no., type, no. people per cluster)* |  |
| **No. randomised per group**  *(specify whether no. people or clusters)* | Intervention: 49 patients, 30 patients completed the study.  Control: 51 patients, 28 patients completed the study. |
| **No. missing**  *(if overall, e.g. exclusions & withdrawals, whether or not missing from analysis)* | Intervention: 19 patients (discontinue; n=1 smoking, n=2 eating mixed rice, n=15 did not show up for their final visits for different reasons including travel out of state or to China or medical reasons, n=8 withdrew because dislike the taste of brown rice).  Control: 23 patients (n=1 smoking, n=5 eating mixed rice, n=2 HbA1c > 6.6%, n=15 did not show up for their final visits for different reasons including travel out of state or to China or medical reasons).  58 patients completed the study, however only 57 patients were reported in the study result. Reported brown rice n=28, white rice n=29. |
| **Reasons missing** | Intervention: 2 patients (not stated)  Control: not stated |
| **Baseline imbalances** |  |
| **Age** | Mean age 55±9 in brown rice group, mean age 50±9 in white rice group. |
| **Sex (proportion)** | 64% female in brown rice group, 69% female in white rice group. |
| **Race/Ethnicity** | Chinese American |
| **Other relevant sociodemographics** |  |
| **Subgroups measured** *(eg split by age or sex)* |  |
| **Subgroups reported** | nil |

Section 2. Risk of bias assessment

We recommend you refer to and use the method described in the Cochrane Handbook.

This section is completed by two reviewers. State initials: (i)AFAR (ii) NMN

| **Domain** | **Risk of bias** | | | **Support for judgement**  *(include direct quotes where available with explanatory comments)* | **Location in text or source** *(page, table)* |
| --- | --- | --- | --- | --- | --- |
|  | Low | High | Unclear |  |  |
| **Random sequence generation**  *(selection bias)* |  |  | Unclear | Quote: “After signing informed consent, subjects were randomized to either continue their regular intake of white rice or change to brown rice for the next 12 weeks” | Page e16 |
| **Allocation concealment**  *(selection bias)* |  | High |  | Quote: “Subjects were encouraged to prepare rice items in their daily meals with the food items provided for the  duration of the study and they were also advised not to change their usual patterns of cooking and eating.” | Page e16 |
| **Blinding of participants and personnel**  *(performance bias)* |  | High |  | Quote: “Subjects were encouraged to prepare rice items in their daily meals with the food items provided for the  duration of the study and they were also advised not to change their usual patterns of cooking and eating.” | Page e16 |
| **Blinding of outcome assessment**  *(detection bias)* |  |  | Unclear | Comment: all the anthropometric measurement and biochemical parameters were done in laboratory but does not mentioned whether the technicians were blinded or not | Page e16 |
| **Incomplete outcome data**  *(attrition bias)* |  | High |  | Comment: 58 patients completed the study, however only 57 patients were reported in the study result. | Page e17, table 2 |
| **Selective outcome reporting**  *(reporting bias)* |  | High |  | Quote: “Linear regression analysis was used to examine the potential effect of weight loss on other changes associated with the brown rice intervention” | Page e17, 2.6 statistical analysis |
| **Other bias** |  |  | Unclear |  |  |

Random sequence generation = Process used to assign people into intervention and control groups

Allocation concealment = Process used to prevent foreknowledge of group assignment in a RCT

Blinding of participants and personnel = Presence or absence of blinding for participants and health personnel

Blinding of outcome assessment = presence or absence of blinding for assessment of outcome

Incomplete outcome data = application of intention-to-treat analysis is one in which all the participants in a trial are analysed according to the intervention to which they were allocated

Selective outcome reporting = Selection of a subset of the original variables recorded

***Section 3. Intervention groups***

This section is completed by two reviewers. State initials: (i)AFAR (ii) NMN

| **Outcomes relevant to your review**  *(Copy and paste from ‘Types of outcome measures’)* | **Reported in paper**  *(Yes / No)* | **Outcome definition** *(with diagnostic criteria if relevant)* | **Unit of measurement & tool**  *(if relevant)* | **Reanalysis required?** *(specify)* |
| --- | --- | --- | --- | --- |
| HbA1c | Yes |  | % |  |
| Fasting blood glucose | Yes |  | mg/dL |  |
| Body weight | Yes |  | kg |  |
| Waist circumference | Yes |  | cm |  |
| Blood pressure | Yes |  | mmHg |  |
| LDL-cholesterol | Yes |  | mg/dL |  |
| HDL-cholesterol | Yes |  | mg/dL |  |

***Section 4. Data and analysis***

| **DICHOTOMOUS OUTCOME** | Intervention group | | Control group | |
| --- | --- | --- | --- | --- |
|  | Number of events | Number of participants | Number of events | Number of participants |
|  |  |  |  |  |
|  |  |  |  |  |
|  |  |  |  |  |
|  |  |  |  |  |
|  |  |  |  |  |
|  |  |  |  |  |

State details if outcomes were only described in text or figures.

| **CONTINUOUS OUTCOME** | Unit of measurement | Intervention group | | Control group | |
| --- | --- | --- | --- | --- | --- |
|  |  | n | Mean (SD) | n | Mean (SD) |
| HbA1c | % | 28 | 5.9 (0.2) | 29 | 5.8 (0.2) |
| Fasting blood glucose | mg/dL | 28 | 93 (9) | 29 | 89 (7) |
| Body weight | kg | 28 | 63.4 (8) | 29 | 63.8 (10) |
| Waist circumference | cm | 28 | 82 (6) | 29 | 84 (8) |
| Blood pressure | mmHg | 28 | 114 (13)  72 (6) | 29 | 118 (18)  76 (8) |
| LDL-cholesterol | mg/dL | 28 | 98 (24) | 29 | 108 (29) |
| HDL-cholesterol | mg/dL | 28 | 52 (12) | 29 | 54 (14) |

State details if outcomes were only described in text or figures.

***Section 5. Other information***

|  | **Description as stated in paper** |
| --- | --- |
| **Key conclusions of study authors** | Whole grain-rich diet is associated with reduction of body weight and blood pressure and  reduced markers of inflammation. In summary, substituting brown rice for white rice in a population with high daily consumption of rice has a beneficial effect in improving their metabolic risk factors. |
| **Results that you calculated using a formula** |  |
| **References to other relevant studies**  *(Did this report include any references to unpublished data from potentially eligible trials not already identified for this review? If yes, give list contact name and details)* |  |
| **Correspondence required for further study information** *(from whom, what and when)* |  |

**Sources:**

Higgins JPT, Green S (editors). Cochrane Handbook for Systematic Reviews of Interventions Version 5.1.0 [updated March 2011]. The Cochrane Collaboration, 2011.Available from www.cochrane-handbook.org.
