# Supplementary material for: The effect of a brown-rice diets on glycemic control and metabolic parameters in prediabetes and type 2 diabetes mellitus: a meta-analysis of randomized controlled trials and controlled clinical trials
Source: PeerJ. 2021 May 26;9:e11291. doi: 10.7717/peerj.11291 (PMC8164413; doi:10.7717/peerj.11291)
Supplement: Supplemental Information 8 [file peerj-09-11291-s008.doc]

***Study Eligibility & Data Collection Form***

***General Information***

| **Study ID**  *(e.g. author name, year)* | Zhang 2017 |
| --- | --- |
| **Form completed by** | Anis Farhanah Abdul Rahim |
| **Study author contact details** | anisfar89@gmail.com |
| **Publication type**  *(e.g. full report, abstract, letter)* | Full report |
| **List of included publications** |  |
| **References of similar trial*** |  |

*This is when the authors published the same study in several reports. All these references to a similar trial should be linked under one *Study ID* in RevMan.

***Study eligibility***

|  | Yes | No | Unclear | Further details |
| --- | --- | --- | --- | --- |
| **RCT/Quasi/CCT** | ***/*** |  |  |  |
| **Relevant participants** | ***/*** |  |  |  |
| **Relevant interventions** | ***/*** |  |  |  |
| **Relevant outcomes*** | ***/*** |  |  |  |

*Include only if the presence of outcomes form the inclusion criterion

If the above answers are ‘YES’, proceed to Section 1.

If any of the above answers are ‘NO*’, record below the information for ‘Excluded studies’

| Reason(s) for exclusion |
| --- |
|  |

Section 1. Characteristics of included studies

This section is to be completed by only one reviewer. State initials: ……

| **METHODS** | **Descriptions as stated in paper** |
| --- | --- |
| **Aim of study** *(e.g. efficacy, equivalence, pragmatic)* | To investigate the effect of replacing WR with BR on metabolic risk factors |
| **Design** *(e.g. parallel, crossover, cluster)* | Parallel, randomized controlled trial |
| **Unit of allocation**  *(by individuals, cluster/ groups or body parts)* |  |
| **Start & end dates** | Not stated |
| **Total study duration** | 16 weeks |
| **Sources of funding**  *(including role of funders)* | Not stated |
| **Possible conflicts of interest**  *(for study authors)* | Not stated |

| **PARTICIPANTS** | **Description**  *(include information for each intervention or comparison group)* |
| --- | --- |
| **Population description**  *(Company/companies; occupation)* | Faculty and staff members with metabolic syndrome |
| **Setting**  *(including location (city, state, country) and single centre / multicenter)* | A large university in Shanghai |
| **Inclusion criteria** | Metabolic syndrome based on the updated National Cholesterol Education Program Adult Treatment Panel III criteria for Asian Americans:  1) waist circumference ≥ 90 cm in men or ≥ 80 cm in women (defined as central obesity);  2) TG ≥ 1.7 mmol/L (defined as elevated TG);  3) HDL cholesterol < 1.03 mmol/L in men or < 1.30 mmol/L in women (defined as reduced HDL cholesterol);  4) blood pressure ≥ 130/85 mm Hg (defined as elevated blood pressure), previously diagnosed hypertension, or using antihypertensive medications; or  5) fasting glucose ≥ 5.6 mmol/L (defined as elevated fasting  glucose), previously diagnosed diabetes, or using hypoglycemic agents |
| **Exclusion criteria** | Individuals with a history of severe kidney disease, cardiovascular disease, stroke, cancer, or psychological disorders as well as pregnant or lactating women were excluded. |
| **Method of recruitment of participants** *(e.g. phone, mail, clinic patients, voluntary)* | Mail |
| **Total no. randomised** | 202 participants were randomly assigned (stratified by sex and 5-y age category) |
| **Clusters**  *(if applicable, no., type, no. people per cluster)* |  |
| **No. randomised per group**  *(specify whether no. people or clusters)* | Intervention: 101 participants  Control: 101 participants |
| **No. missing**  *(if overall, e.g. exclusions & withdrawals, whether or not missing from analysis)* | Intervention: 3 participants dropped out  Control: 6 participants withdrew |
| **Reasons missing** | Intervention: busy schedule (n = 1) or intervention-unrelated heart disease (n = 2)  Control: busy schedule (n = 4), loss of interest (n = 1), or intervention-unrelated stroke (n = 1). |
| **Baseline imbalances** | Nil |
| **Age** | Mean age of 49 years old |
| **Sex (proportion)** | 47 out of 101 participants are female (intervention group)  47 out of 101 participants are female (control group) |
| **Race/Ethnicity** | Chinese |
| **Other relevant sociodemographics** | University faculty members and staff |
| **Subgroups measured** *(eg split by age or sex)* |  |
| **Subgroups reported** | Participants with and without MetS, diabetes, hypertension, dyslipidemia, and central obesity at baseline |

Section 2. Risk of bias assessment

We recommend you refer to and use the method described in the Cochrane Handbook.

This section is completed by two reviewers. State initials: (i)AFAR (ii) NMN

| **Domain** | **Risk of bias** | | | **Support for judgement**  *(include direct quotes where available with explanatory comments)* | **Location in text or source** *(page, table)* |
| --- | --- | --- | --- | --- | --- |
|  | Low | High | Unclear |  |  |
| **Random sequence generation**  *(selection bias)* |  | High |  | Quotes: “a total of 202 participants was randomly assigned (stratified by sex and 5-y age category) to a WR or BR group” | Page 1686 |
| **Allocation concealment**  *(selection bias)* |  | High |  | Quotes: “The cooked rice was packaged into 225-g servings (equivalent to 100 g uncooked rice) and provided to the participants at designated campus cafeterias during the lunch hour from Mondays to Fridays. The participants took the cooked rice home for dinner and meals on Saturdays” | Page 1686 |
| **Blinding of participants and personnel**  *(performance bias)* |  | High |  | Quote: “Although it was not possible for participants to be unaware of their assignment due to the obvious differences in appearance and texture between BR and WR, all the researchers not directly in contact with study participants (dietitians, laboratory technicians, and statisticians) were unaware of group allocations” | Page 1686 |
| **Blinding of outcome assessment**  *(detection bias)* | Low |  |  | Quote: “all the researchers not directly in contact with study participants (dietitians, laboratory technicians, and statisticians) were unaware of group allocations” | Page 1686 |
| **Incomplete outcome data**  *(attrition bias)* |  | High |  | Quote: “Six participants in the WR group withdrew due to a busy schedule (n = 4), loss of interest (n = 1), or intervention-unrelated stroke (n = 1). Three participants in the BR arm dropped out because of a busy schedule (n = 1) or intervention-unrelated heart disease (n = 2). Thus, a total of 193 participants (95.5%) completed the 16-wk intervention.”  Comment: 193 participants completed the intervention but total of 202 were reported in the result | Page 1687 |
| **Selective outcome reporting**  *(reporting bias)* |  | High |  | Quote: “Body weight decreased  significantly from baseline in the BR group”  Comment: Body weight difference between BR and WR not stated | Page 1688 |
| **Other bias** |  |  |  |  |  |

Random sequence generation = Process used to assign people into intervention and control groups

Allocation concealment = Process used to prevent foreknowledge of group assignment in a RCT

Blinding of participants and personnel = Presence or absence of blinding for participants and health personnel

Blinding of outcome assessment = presence or absence of blinding for assessment of outcome

Incomplete outcome data = application of intention-to-treat analysis is one in which all the participants in a trial are analysed according to the intervention to which they were allocated

Selective outcome reporting = Selection of a subset of the original variables recorded

***Section 3. Intervention groups***

This section is completed by two reviewers. State initials: (i)AFAR (ii) NMN

| **Outcomes relevant to your review**  *(Copy and paste from ‘Types of outcome measures’)* | **Reported in paper**  *(Yes / No)* | **Outcome definition** *(with diagnostic criteria if relevant)* | **Unit of measurement & tool**  *(if relevant)* | **Reanalysis required?** *(specify)* |
| --- | --- | --- | --- | --- |
| HbA1c | Yes |  | %, using an automated immunoassay (Roche Diagnostics) |  |
| Fasting blood glucose | Yes |  | mmol/L, performed on an automatic analyzer (Hitachi 7080) within 2 consecutive days |  |
| Body weight | No |  | kg, measured in light indoor clothing without shoes to the nearest 0.1 kg |  |
| Waist circumference | Yes |  | cm, measured at the mid-point between the lowest rib and the iliac crest to the nearest 0.1 cm after inhalation and exhalation |  |
| Blood pressure | Yes |  | mmHg, electronic blood pressure monitor (Omron HEM-7000, Dalian) |  |
| LDL-cholesterol | Yes |  | mmol/L, performed on an automatic analyzer (Hitachi 7080) within 2 consecutive days |  |
| HDL-cholesterol | Yes |  | mmol/L, performed on an automatic analyzer (Hitachi 7080) within 2 consecutive days |  |

***Section 4. Data and analysis***

| **DICHOTOMOUS OUTCOME** | Intervention group | | Control group | |
| --- | --- | --- | --- | --- |
|  | Number of events | Number of participants | Number of events | Number of participants |
|  |  |  |  |  |
|  |  |  |  |  |
|  |  |  |  |  |
|  |  |  |  |  |
|  |  |  |  |  |
|  |  |  |  |  |

State details if outcomes were only described in text or figures.

| CONTINUOUS OUTCOME | Unit of measurement | Intervention group | | Control group | |
| --- | --- | --- | --- | --- | --- |
|  |  | n | Mean (SD) | n | Mean (SD) |
| HbA1c | % | 101 | Mean (95% CI): 0.13(-0.03 to 0.22) | 101 | Mean (95% CI): 0.20(0.04 to 0.36) |
| Fasting blood glucose | mmol/L | 101 | Mean (95% CI): 0.01(-0.21 to 0.22) | 101 | Mean (95% CI): -0.16(-0.45 to 0.13) |
| Body weight | kg |  |  |  |  |
| Waist circumference | cm | 101 | Mean (95% CI): -0.27(-0.86 to 0.31) | 101 | Mean (95% CI): -0.37(-0.78 to 0.04) |
| Blood pressure | mmHg | 101 | SBP: Mean (95% CI): -5.39(-7.49 to -3.29)  DBP: Mean (95% CI): -3.29(-5.25 to -2.58) | 101 | SBP: Mean (95% CI): -4.78 (-6.88 to -2.68)  DBP: Mean (95% CI): -3.82 (-5.13 to -2.51) |
| LDL-cholesterol | mmol/L | 101 | Mean (95% CI): -0.03 (-0.21 to 0.16) | 101 | Mean (95% CI): -0.33 (-0.52 to -0.14) |
| HDL-cholesterol | mmol/L | 101 | Mean (95% CI): -0.03 (-0.08 to 0.02) | 101 | Mean (95% CI): -0.12 (-0.18 to -0.06) |

State details if outcomes were only described in text or figures.

***Section 5. Other information***

|  | **Description as stated in paper** |
| --- | --- |
| **Key conclusions of study authors** | Substituting white rice with brown rice for 16 weeks does not substantially affect metabolic risk factors in middle-aged Chinese men and women with diabetes  or a high risk for diabetes |
| **Results that you calculated using a formula** | Means (95% CI) converted to means (SD) |
| **References to other relevant studies**  *(Did this report include any references to unpublished data from potentially eligible trials not already identified for this review? If yes, give list contact name and details)* |  |
| **Correspondence required for further study information** *(from whom, what and when)* |  |

**Sources:**

Higgins JPT, Green S (editors). Cochrane Handbook for Systematic Reviews of Interventions Version 5.1.0 [updated March 2011]. The Cochrane Collaboration, 2011.Available from www.cochrane-handbook.org.
