# Supplementary material for: The effect of a brown-rice diets on glycemic control and metabolic parameters in prediabetes and type 2 diabetes mellitus: a meta-analysis of randomized controlled trials and controlled clinical trials
Source: PeerJ. 2021 May 26;9:e11291. doi: 10.7717/peerj.11291 (PMC8164413; doi:10.7717/peerj.11291)
Supplement: Supplemental Information 9 [file peerj-09-11291-s009.docx]

***Study Eligibility & Data Collection Form***

***General Information***

| **Study ID**  *(e.g. author name, year)* | Nakayama 2017 |
| --- | --- |
| **Form completed by** | Anis Farhanah Abdul Rahim |
| **Study author contact details** | anisfar89@gmail.com |
| **Publication type**  *(e.g. full report, abstract, letter)* | Full report |
| **List of included publications** |  |
| **References of similar trial*** |  |

*This is when the authors published the same study in several reports. All these references to a similar trial should be linked under one *Study ID* in RevMan.

***Study eligibility***

|  | Yes | No | Unclear | Further details |
| --- | --- | --- | --- | --- |
| **RCT/Quasi/CCT** | ***/*** |  |  |  |
| **Relevant participants** | ***/*** |  |  |  |
| **Relevant interventions** | ***/*** |  |  |  |
| **Relevant outcomes*** | ***/*** |  |  |  |

*Include only if the presence of outcomes form the inclusion criterion

If the above answers are ‘YES’, proceed to Section 1.

If any of the above answers are ‘NO*’, record below the information for ‘Excluded studies’

| Reason(s) for exclusion |
| --- |
|  |

Section 1. Characteristics of included studies

This section is to be completed by only one reviewer. State initials: AFAR

| **METHODS** | **Descriptions as stated in paper** |
| --- | --- |
| **Aim of study** *(e.g. efficacy, equivalence, pragmatic)* | evaluated the effect of GBR intake for 8 weeks on glycemic control in outpatients with diabetes mellitus. |
| **Design** *(e.g. parallel, crossover, cluster)* | open-label randomized crossover study in outpatients with type 2 diabetes |
| **Unit of allocation**  *(by individuals, cluster/ groups or body parts)* |  |
| **Start & end dates** | Between August 2015 and June 2016 |
| **Total study duration** | 8 weeks |
| **Sources of funding**  *(including role of funders)* | Not stated |
| **Possible conflicts of interest**  *(for study authors)* | No conflict of interest declared |

| **PARTICIPANTS** | **Description**  *(include information for each intervention or comparison group)* |
| --- | --- |
| **Population description**  *(Company/companies; occupation)* | patients with type 2 diabetes were recruited at the outpatient clinic of St Marianna University Hospital (Kawasaki, Japan). |
| **Setting**  *(including location (city, state, country) and single centre / multicenter)* | outpatient clinic of St Marianna University Hospital (Kawasaki, Japan). |
| **Inclusion criteria** | (1) an age ⩾20 years,  (2) stable HbA1c for 6 months (HbA1>46.0 and<8.9 with ΔHbA1c<0.5%) and  (3) treatment with multiple daily insulin injections with or without oral hypoglycemic agents |
| **Exclusion criteria** | (1) an age ⩾75 years,  (2) type 1 diabetes,  (3) severe renal dysfunction (estimated glomerular filtration rate <30 ml− 1 min− 1 per 1.73 m− 2),  (4) women who were pregnant, possibly pregnant, planned to  become pregnant, or were breastfeeding and  (5) patients who were ineligible for the study by the attending doctor for other reasons. The treatment of the patients, including oral antidiabetic agents and insulin doses, was not changed throughout the study period. |
| **Method of recruitment of participants** *(e.g. phone, mail, clinic patients, voluntary)* | Outpatient clinic patients with type 2 diabetes mellitus |
| **Total no. randomised** | 18 participants were randomly assigned to two groups |
| **Clusters**  *(if applicable, no., type, no. people per cluster)* |  |
| **No. randomised per group**  *(specify whether no. people or clusters)* | WR n=9  BR n=9 |
| **No. missing**  *(if overall, e.g. exclusions & withdrawals, whether or not missing from analysis)* | WR: discontinued intervention for personal reason (n=1)  BR: discontinued intervention, hospitalised for colorectal ca (n=1) |
| **Reasons missing** | WR: discontinued intervention for personal reason (n=1)  BR: discontinued intervention, hospitalised for colorectal ca (n=1) |
| **Baseline imbalances** | Nil |
| **Age** | Mean age of 64 + 8.8 (range 45-74yo) |
| **Sex (proportion)** | 12 men and 4 women |
| **Race/Ethnicity** | Not mentioned |
| **Other relevant sociodemographics** |  |
| **Subgroups measured** *(eg split by age or sex)* |  |
| **Subgroups reported** |  |

Section 2. Risk of bias assessment

We recommend you refer to and use the method described in the Cochrane Handbook.

This section is completed by two reviewers. State initials: (i)AFAR (ii) AMZ

| **Domain** | **Risk of bias** | | | **Support for judgement**  *(include direct quotes where available with explanatory comments)* | **Location in text or source** *(page, table)* |
| --- | --- | --- | --- | --- | --- |
|  | Low | High | Unclear |  |  |
| **Random sequence generation**  *(selection bias)* |  |  | Unclear | Quotes: “After eating WR twice daily for a 1-week observation period, the subjects were randomly assigned to two groups. One group ate GBR twice a day for 8 weeks as a staple food, after which they switched to WR for the next 8 weeks, whereas the other group ate WR first and then GBR.” | Page 2 |
| **Allocation concealment**  *(selection bias)* |  | High |  | Comment: no allocation concealment mentioned. However, there is difficulty to blind the rice types. | Page 2 |
| **Blinding of participants and personnel**  *(performance bias)* |  | High |  | Quote: “difficulty of blinding the different types of rice.”  Comment: this study used an open-label crossover design because of the difficulty of blinding the different types of rice. | Page 4 |
| **Blinding of outcome assessment**  *(detection bias)* | Low |  |  | Comment: blinding of outcome assessment mentioned but because he primary end point of this study was the change of HbA1c from baseline which were done by machine, the detection bias is assessed as being low risk | Page 2 |
| **Incomplete outcome data**  *(attrition bias)* | Low |  |  | Quote: “Among the 18 subjects registered in this study, 2 were excluded from analysis because of personal problems (n = 1) and emergency hospitalization for colorectal cancer (n = 1). The other 16  subjects (12 men and 4 women) completed the study and formed  the per protocol set for analyses” | Page 2 |
| **Selective outcome reporting**  *(reporting bias)* | Low |  |  | Comment: all primary end point and secondary end point were measured | Page 2-3 |
| **Other bias** | Low |  |  | No other bias detected |  |

Random sequence generation = Process used to assign people into intervention and control groups

Allocation concealment = Process used to prevent foreknowledge of group assignment in a RCT

Blinding of participants and personnel = Presence or absence of blinding for participants and health personnel

Blinding of outcome assessment = presence or absence of blinding for assessment of outcome

Incomplete outcome data = application of intention-to-treat analysis is one in which all the participants in a trial are analysed according to the intervention to which they were allocated

Selective outcome reporting = Selection of a subset of the original variables recorded

***Section 3. Intervention groups***

This section is completed by two reviewers. State initials: (i)AFAR (ii) AMZ

| **Outcomes relevant to your review**  *(Copy and paste from ‘Types of outcome measures’)* | **Reported in paper**  *(Yes / No)* | **Outcome definition** *(with diagnostic criteria if relevant)* | **Unit of measurement & tool**  *(if relevant)* | **Reanalysis required?** *(specify)* |
| --- | --- | --- | --- | --- |
| HbA1c | Yes |  | Unit %, tool not mentioned |  |
| Fasting blood glucose | No |  |  |  |
| Body weight | No |  |  |  |
| Waist circumference | No |  |  |  |
| Blood pressure | No |  |  |  |
| LDL-cholesterol | No |  |  |  |
| HDL-cholesterol | No |  |  |  |

***Section 4. Data and analysis***

| **DICHOTOMOUS OUTCOME** | Intervention group | | Control group | |
| --- | --- | --- | --- | --- |
|  | Number of events | Number of participants | Number of events | Number of participants |
|  |  |  |  |  |
|  |  |  |  |  |
|  |  |  |  |  |
|  |  |  |  |  |
|  |  |  |  |  |
|  |  |  |  |  |

State details if outcomes were only described in text or figures.

| CONTINUOUS OUTCOME | Unit of measurement | Intervention group | | Control group | |
| --- | --- | --- | --- | --- | --- |
|  |  | n | Mean (SD) | n | Mean (SD) |
| HbA1c | % | 8 | 7.1 + 0.5% | 8 | 7.5 + 0.9% |
|  |  |  |  |  |  |

State details if outcomes were only described in text or figures.

***Section 5. Other information***

|  | **Description as stated in paper** |
| --- | --- |
| **Key conclusions of study authors** | In conclusion, eating GBR twice a day for 8 weeks was well tolerated with respect to palatability and led to sustained improvement of glycemic control in patients with type 2 diabetes. |
| **Results that you calculated using a formula** | Means (95% CI) converted to means (SD) |
| **References to other relevant studies**  *(Did this report include any references to unpublished data from potentially eligible trials not already identified for this review? If yes, give list contact name and details)* |  |
| **Correspondence required for further study information** *(from whom, what and when)* |  |

**Sources:**

Higgins JPT, Green S (editors). Cochrane Handbook for Systematic Reviews of Interventions Version 5.1.0 [updated March 2011]. The Cochrane Collaboration, 2011.Available from www.cochrane-handbook.org.
