# Supplementary material for: The effect of a brown-rice diets on glycemic control and metabolic parameters in prediabetes and type 2 diabetes mellitus: a meta-analysis of randomized controlled trials and controlled clinical trials
Source: PeerJ. 2021 May 26;9:e11291. doi: 10.7717/peerj.11291 (PMC8164413; doi:10.7717/peerj.11291)
Supplement: Supplemental Information 10 [file peerj-09-11291-s010.doc]

***Study Eligibility & Data Collection Form***

***General Information***

| **Study ID**  *(e.g. author name, year)* | Hsu 2008 |
| --- | --- |
| **Form completed by** | Anis Farhanah Abdul Rahim |
| **Study author contact details** | anisfar89@gmail.com |
| **Publication type**  *(e.g. full report, abstract, letter)* | Full report |
| **List of included publications** |  |
| **References of similar trial*** |  |

*This is when the authors published the same study in several reports. All these references to a similar trial should be linked under one *Study ID* in RevMan.

***Study eligibility***

|  | Yes | No | Unclear | Further details |
| --- | --- | --- | --- | --- |
| **RCT/Quasi/CCT** | ***/*** |  |  |  |
| **Relevant participants** | ***/*** |  |  |  |
| **Relevant interventions** | ***/*** |  |  |  |
| **Relevant outcomes*** | ***/*** |  |  |  |

*Include only if the presence of outcomes form the inclusion criterion

If the above answers are ‘YES’, proceed to Section 1.

If any of the above answers are ‘NO*’, record below the information for ‘Excluded studies’

| Reason(s) for exclusion |
| --- |
|  |

Section 1. Characteristics of included studies

This section is to be completed by only one reviewer. State initials: AFAR

| **METHODS** | **Descriptions as stated in paper** |
| --- | --- |
| **Aim of study** *(e.g. efficacy, equivalence, pragmatic)* | To ascertain the clinical usefulness of a diet including PGBR on blood glucose management in patients with impaired fasting glucose or type 2 diabetes |
| **Design** *(e.g. parallel, crossover, cluster)* | Crossover, randomized controlled trial |
| **Unit of allocation**  *(by individuals, cluster/ groups or body parts)* |  |
| **Start & end dates** | Not stated |
| **Total study duration** | 6 weeks |
| **Sources of funding**  *(including role of funders)* | Not stated |
| **Possible conflicts of interest**  *(for study authors)* | Not stated |

| **PARTICIPANTS** | **Description**  *(include information for each intervention or comparison group)* |
| --- | --- |
| **Population description**  *(Company/companies; occupation)* | Free living subjects with type 2 diabetes (6 men 5 women) |
| **Setting**  *(including location (city, state, country) and single centre / multicenter)* | Li Shin Hospital in Taiwan |
| **Inclusion criteria** | Inclusion criteria, fasting blood glucose >110mg/dL |
| **Exclusion criteria** | Not stated |
| **Method of recruitment of participants** *(e.g. phone, mail, clinic patients, voluntary)* | Subjects were recruited through physician at Li Shin Hospital (Pingjen, Taiwan) |
| **Total no. randomised** | 11 free-living subjects with type 2 diabetes were randomly allocated to two experimental periods of 6 week of WR or PGBR diet in a crossover design (first intervention period: 0-6wk; second intervention period: 8 to 14 wk) |
| **Clusters**  *(if applicable, no., type, no. people per cluster)* |  |
| **No. randomised per group**  *(specify whether no. people or clusters)* | Group 1 (WR) n=6  Group 2 (PGBR) n=5 |
| **No. missing**  *(if overall, e.g. exclusions & withdrawals, whether or not missing from analysis)* | Not stated no of dropout (? No dropout) |
| **Reasons missing** |  |
| **Baseline imbalances** | Nil |
| **Age** | Mean age 51.5+16.2y (range 27-72y) |
| **Sex (proportion)** | 6 men 5 women |
| **Race/Ethnicity** | Free-living in Taiwan |
| **Other relevant sociodemographics** |  |
| **Subgroups measured** *(eg split by age or sex)* |  |
| **Subgroups reported** |  |

Section 2. Risk of bias assessment

We recommend you refer to and use the method described in the Cochrane Handbook.

This section is completed by two reviewers. State initials: (i)AFAR (ii) AMZ

| **Domain** | **Risk of bias** | | | **Support for judgement**  *(include direct quotes where available with explanatory comments)* | **Location in text or source** *(page, table)* |
| --- | --- | --- | --- | --- | --- |
|  | Low | High | Unclear |  |  |
| **Random sequence generation**  *(selection bias)* |  |  | Unclear | Quotes: “the participants were randomly allocated to two experimental periods of 6 week of WR or PGBR diet in a crossover design (first intervention period: 0-6wk; second intervention period: 8 to 14 wk)” | Page 164 |
| **Allocation concealment**  *(selection bias)* |  | High |  | Quotes: “during the 2 intervention periods, subjects were instructed to consume either WR or PGBR as the staple food” | Page 164 |
| **Blinding of participants and personnel**  *(performance bias)* |  | High |  | Quote: “during the 2 intervention periods, subjects were instructed to consume either WR or PGBR as the staple food”  Comment: It is difficult to blind the rice types. | Page 164 |
| **Blinding of outcome assessment**  *(detection bias)* | Low |  |  | Quote: “laboratory technologists were blinded to the identity of subjects and intervention status, and the person in charge of statistical analyses was blinded to the same information until the time of data analysis” | Page 165 |
| **Incomplete outcome data**  *(attrition bias)* | Low |  |  | All randomized sample were analysed | Page 164 |
| **Selective outcome reporting**  *(reporting bias)* | Low |  |  | All mentioned outcomes were measured | Page 164 |
| **Other bias** | Low |  |  | No other bias detected |  |

Random sequence generation = Process used to assign people into intervention and control groups

Allocation concealment = Process used to prevent foreknowledge of group assignment in a RCT

Blinding of participants and personnel = Presence or absence of blinding for participants and health personnel

Blinding of outcome assessment = presence or absence of blinding for assessment of outcome

Incomplete outcome data = application of intention-to-treat analysis is one in which all the participants in a trial are analysed according to the intervention to which they were allocated

Selective outcome reporting = Selection of a subset of the original variables recorded

***Section 3. Intervention groups***

This section is completed by two reviewers. State initials: (i)AFAR (ii) AMZ

| **Outcomes relevant to your review**  *(Copy and paste from ‘Types of outcome measures’)* | **Reported in paper**  *(Yes / No)* | **Outcome definition** *(with diagnostic criteria if relevant)* | **Unit of measurement & tool**  *(if relevant)* | **Reanalysis required?** *(specify)* |
| --- | --- | --- | --- | --- |
| HbA1c | No |  |  |  |
| Fasting blood glucose | Yes but no post assessment data prior to crossover |  |  |  |
| Body weight | Yes |  | kg |  |
| Waist circumference | No |  |  |  |
| Blood pressure | Yes |  | mmHg, tool used is not stated |  |
| LDL-cholesterol | No |  |  |  |
| HDL-cholesterol | Yes |  | Mg/dL, measured using the free glycocerol diminishing method |  |

***Section 4. Data and analysis***

| **DICHOTOMOUS OUTCOME** | Intervention group | | Control group | |
| --- | --- | --- | --- | --- |
|  | Number of events | Number of participants | Number of events | Number of participants |
|  |  |  |  |  |
|  |  |  |  |  |
|  |  |  |  |  |
|  |  |  |  |  |
|  |  |  |  |  |

State details if outcomes were only described in text or figures.

| CONTINUOUS OUTCOME | Unit of measurement | Intervention group | | Control group | |
| --- | --- | --- | --- | --- | --- |
|  |  | n | Mean (SD) | n | Mean (SD) |
| Body weight | kg | 5 | 64.6+3.0 | 6 | 65.9+6.1 |
| Blood pressure | mmHg | 5 | SBP: 118.3+7.0  DBP: 69.0+3.8 | 6 | SBP: 125.6+8.5  DBP: 75.2+3.0 |
| HDL-cholesterol | Mg/dL | 5 | 46.7+3.8 | 6 | 63.2+4.2 |

State details if outcomes were only described in text or figures.

***Section 5. Other information***

|  | **Description as stated in paper** |
| --- | --- |
| **Key conclusions of study authors** | Consumption of PGBR significantly improved levels of blood lipids |
| **Results that you calculated using a formula** | Mean+SE |
| **References to other relevant studies**  *(Did this report include any references to unpublished data from potentially eligible trials not already identified for this review? If yes, give list contact name and details)* |  |
| **Correspondence required for further study information** *(from whom, what and when)* |  |

**Sources:**

Higgins JPT, Green S (editors). Cochrane Handbook for Systematic Reviews of Interventions Version 5.1.0 [updated March 2011]. The Cochrane Collaboration, 2011.Available from www.cochrane-handbook.org.
